# Spatial transcriptomics and *in situ* immune cell profiling of the host ectocervical landscape of HIV infected Kenyan sex working women

**DOI:** 10.3389/fimmu.2024.1483346

**Published:** 2024-12-02

**Authors:** Mathias Franzén Boger, Vilde Kaldhusdal, Anna Pascual-Reguant, Sandy Kroh, Ralf Uecker, Adam D. Burgener, Julie Lajoie, Kenneth Omollo, Joshua Kimani, Keith R. Fowke, Anja E. Hauser, Annelie Tjernlund, Kristina Broliden

**Affiliations:** ^1^ Department of Medicine Solna, Division of Infectious Diseases, Karolinska Institutet, Division of Infectious Diseases, Karolinska University Hospital, Center for Molecular Medicine, Stockholm, Sweden; ^2^ Department of Rheumatology and Clinical Immunology, Charité–Universitätsmedizin Berlin, Berlin, Germany; ^3^ Immune Dynamics, Deutsches Rheuma-Forschungzentrum (DRFZ), Leibniz Insititute, Berlin, Germany; ^4^ Spatial Genomics, Centre Nacional d’Anàlisi Genòmica, Barcelona, Spain; ^5^ Center for Global Health and Diseases, Department of Pathology, Case Western Reserve University, Cleveland, OH, United States; ^6^ Department of Obstetrics and Gynecology, University of Manitoba, Winnipeg, MB, Canada; ^7^ Department of Medical Microbiology and Infectious Diseases, University of Manitoba, Winnipeg, MB, Canada; ^8^ Department of Medical Microbiology, University of Nairobi, Nairobi, Kenya; ^9^ Partners for Health and Development in Africa, Nairobi, Kenya; ^10^ Department of Community Health Sciences, University of Manitoba, Winnipeg, MB, Canada

**Keywords:** HIV, spatial transcriptomics, multi-epitope ligand cartography, mucosal immunology, T cells, B cells, interferon

## Abstract

**Introduction:**

Chronic immune activation is a hallmark of human immunodeficiency virus (HIV) infection that significantly impacts disease pathogenesis. However, in-depth studies characterizing the immunological landscape of the ectocervix during chronic HIV infection remain scarce despite the importance of this tissue site for HIV transmission.

**Methods:**

Ectocervical tissue samples were obtained from antiretroviral-naïve HIV-seropositive and -seronegative Kenyan female sex workers. These samples were assessed by spatial transcriptomics and Gene Set Enrichment Analysis. We further performed multi-epitope ligand cartography (MELC) using an *in situ* staining panel that included 17 markers of primarily T cell–mediated immune responses.

**Results:**

Spatial transcriptomics revealed tissue-wide immune activation encompassing immune responses associated with chronic HIV infection. First, both the epithelial and submucosal compartments showed diverse but significant upregulation of humoral immune responses, as indicated by the expression of several antibody-related genes. Second, an antiviral state–associated cellular immunity was also observed in the HIV-seropositive group, characterized by upregulation of genes involved in interferon signaling across the mucosal tissue and a more spatially restricted mucosal expression of genes related to T cell activity and effector functions relative to the HIV-seronegative group. Additionally, HIV associated structural alterations were evident within both compartments. Downregulated genes across the epithelium were mainly linked to epithelial integrity, with the outer layer involved in terminal differentiation and the inner layer associated with epithelial structure. MELC analysis further revealed a significantly increased ectocervical leukocyte population in HIV-seropositive participants, primarily driven by an increase in CD8^+^ T cells while the CD4^+^ T cell population remained stable. Consistent with our spatial transcriptomics data, T cells from HIV-seropositive participants showed an increased effector phenotype, defined by elevated expression of various granzymes.

**Conclusion:**

By combining spatial transcriptomics and MELC, we identified significant HIV-associated cervical immune activity driven by induction of both T and B cell activity, together with a general antiviral state characterized by sustained interferon induction. These findings underscore that chronic HIV infection is associated with an altered ectocervical mucosal immune landscape years after primary infection. This sheds light on HIV pathogenesis at distant local sites and complements current knowledge on HIV-associated systemic immune activation.

## 1 Introduction

Systemic chronic immune activation is a hallmark feature of human immunodeficiency virus (HIV) infection that plays a pivotal role in the pathogenesis of the disease and progression to acquired immunodeficiency syndrome (AIDS) (1–4). Although the introduction of combined antiretroviral therapy has significantly increased the lifespan of individuals with HIV, such therapies have also exposed the long-term impacts of chronic immune activation, leading to the emergence of several non-AIDS-defining disorders including cardiovascular diseases and non-HIV associated cancers, which are more typically observed in aging populations, in younger individuals living with HIV (5, 6). Simian immunodeficiency virus infection studies in non-human primate models have further revealed the effects of chronic immune activation. These investigations have shown that although both non-pathogenic (including sooty mangabey and African green monkeys), and pathogenic (including several macaque species) non-human primate models sustain high viremia levels and induce acute antiviral responses, non-pathogenic hosts do not show an increased CD4^+^ cell turnover and exhibit an early resolution of acute T cell activation without progression to AIDS-like disease (7–12). The underlying cause of HIV-associated immune activation remains incompletely understood but is thought to be multifactorial and dependent on both intrinsic and extrinsic factors, involving mechanisms that include CD4^+^ T cell depletion, microbial translocation, antigenic stimulation, dysfunctional interferon (IFN) signaling, and an altered balance of T cell subsets (13).

Most HIV infections in women are believed to occur via the cervicovaginal mucosa following sexual intercourse with an infected partner (14, 15). However, the precise location for efficient viral entry within the female reproductive tract (FRT) and the specific mechanisms by which HIV breaches the epithelial barrier to access its target cells remain incompletely understood (16–18). Comprehensive immunological investigations assessing the FRT immune system from *ex vivo* human tissue are logistically challenging and rare in the literature. More commonly, studies have employed cervicovaginal lavage and cytobrush-collected cervical mononuclear cells for this purpose, revealing elevated levels of pro-inflammatory cytokines and dysregulated immune cell populations within the FRT during chronic HIV infection (19–22). Several reports further suggest a crucial function for tissue-resident memory (TRM) T cells in limiting viral infection and dissemination, although their precise role in the pathogenesis and persistence of HIV remains to be fully elucidated (23–25).

In recent years, several cutting-edge techniques, including massive multiplex immunohistochemistry and spatial transcriptomics, have revolutionized our understanding of tissue immunology, enabling the simultaneous visualization of multiple protein markers in tissue sections and identification of high-resolution gene expression patterns, respectively. Together, they offer a powerful approach for exploring the spatial organization and functional dynamics of immune cells within tissues, thus enhancing our understanding of immune-mediated diseases and therapeutic responses. Here, we applied these techniques to explore the transcriptional and cellular landscape of ectocervical tissue samples from Kenyan female sex workers (FSWs) living with chronic HIV infection.

## 2 Materials and methods

### 2.1 Cohort description and sample collection

Study participants included HIV-seropositive (HIV^+^) and -seronegative (HIV^−^) FSWs recruited as a part of a larger longitudinal study from the Pumwani Sex Worker Cohort in Kenya, as previously described (26). Briefly, enrollment inclusion criteria for the larger longitudinal study included actively engaging in sex work, age 18–50 years old, not pregnant or breastfeeding, not menopausal, no history of prior hysterectomy, and willingness to undergo ectocervical biopsy collection. Sampling was aimed at both the follicular and luteal phases based on self-reported time since last menses. Plasma levels of estradiol and progesterone were measured during both phases. Follicular phase samples were measured using electrochemiluminescence immunoassays (Roche Diagnostics, Basel, Switzerland) with a lower limit of detection set at 22 pg/ml and 0.05 ng/ml, respectively, while the Milliplex Map Steroid/Thyroid Hormone Magnetic Bead Panel (Millipore, Merck, Darmstadt, Germany) was used for the luteal phase samples with a lower limit of detection set at 20 pg/ml and 0.09 ng/ml, respectively. All participants were required to test negative for *Chlamydia trachomatis, Neisseria gonorrhoeae, Treponema pallidum*, and *Trichomonas vaginalis* infection at the time of enrollment. *C. trachomatis* and *N. gonorrhoeae* were diagnosed by PCR analysis of urine samples (Roche AMPLICOR, Pleasanton, NJ, USA). The Macro-Vue Rapid Plasma Reagin test (Becton Dickinson, Franklin Lakes, NJ, USA) was used to detect *Treponema pallidum* and *Trichomonas vaginalis* from saline microscopy samples. Bacterial vaginosis was evaluated using Nugent’s score on gram-stained smears, and the molecular microbiome composition was identified by 16S ribosomal RNA sequencing of cervicovaginal lavage samples (27). All participants answered a demographic and behavioral questionnaire and had to abstain from vaginal intercourse for a 2-week healing period after each study sample collection visits. All women were monetarily compensated for a 4-week period to enable healing and underwent testing for the presence of prostate-specific antigen to ensure adherence to the sexual abstinence.

None of the HIV^+^FSWs enrolled in this study had any AIDS-defining illnesses or were on any antiretroviral therapy, as their CD4^+^ cell counts were above the recommended threshold for initiation of treatment at the time of inclusion. FSWs who were HIV^−^ at study onset remained seronegative for 3–6 months after study completion. All study participants had access to medical care, including HIV treatment, as needed. Additionally, study participants received counseling for sexually transmitted infection prevention as well as both male and female condoms.

Two ectocervical tissue biopsies measuring 3 mm^2^, from the superior portion of the ectocervix, were obtained from each participant using Schubert biopsy forceps (B. Braun Aesculap) at both the follicular and luteal phases of the menstrual cycle. The samples had to contain both epithelium and submucosa to be included in the study. Biopsies were snap-frozen and cryopreserved at −80° until use for *in situ* immune staining or spatial transcriptomic analysis.

### 2.2 Ethics statement

This study was performed in accordance with the Helsinki Declaration and was approved by the ethical review boards at the University of Manitoba (HS15280(B2012:043)), the Kenyatta National Hospital and University of Nairobi (P224/04/2012, amendment 2017-04-03), and the regional Ethical Review Board in Stockholm (KI:2018:1306-31). Written informed consent was obtained from all participants.

### 2.3 Sample processing and Visium library preparation

Ectocervical tissue biopsies collected at the follicular phase were sectioned at a thickness of 8 μm using a cryostat and placed within the 6.5-mm^2^ oligo-barcode capture area of the Visium 10x Genomics slide containing thousands of spots measuring 55 μm in diameter. Prior to sequencing and subsequent analysis, the tissue sections were stained by hematoxylin and eosin (H&E) and imaged for visualization and tissue compartmentalization. Library preparation for sequencing was performed according to the manufacturer’s instructions, and RNA sequencing (RNA-seq) was performed using a NovaSeq 6000 flow cell in “SP” mode, with Read 1 set to 28 bases, Index 1 and 2 set to 10 bases each, and Read 2 set to 90 bases. BCL files were converted to FastQ files using the bcl2fastq_v2.20.0.422 from the CASAVA software suite.

### 2.4 Data preprocessing of transcriptional data

The acquired raw reads were processed by the spaceranger command line tool (v.2.1.0, 10x Genomics) and mapped to the human reference genome (*Homo sapiens*, GRCh38-2020-A). For each sample, we then used the load10x_spatial and Read10X_image functions to load and convert the gene expression matrices and tissue images, respectively, into Seurat objects. To ensure the quality of each spot within the samples, we filtered the merged dataset by removing spots containing <100 genes or >25% mitochondrial or hemoglobulin genes. We then identified the ~2000 most variable genes (excluding the *VDJ* genes), which were normalized, scaled and centered using the NormalizeData and ScaleData functions in Seurat (28). Next, the normalized and scaled most variable gene was subjected to principal component analysis (PCA). The normalized dataset was further integrated using Harmony, and unsupervised clustering was performed (dims=30, k.param=15) using a nearest-neighbor approach (kNN). The data were then embedded into a two-dimensional (2D) uniform manifold approximation and projection (UMAP) plot using the initial 50 Harmony vectors as input into the Seurat function RunUMAP. Based on the H&E staining of each tissue section, we then manually outlined the epithelial and submucosal compartments in Affinity Designer 2 (v.2.1.0 Serif Ltd, UK) and used this information to classify each spot as either epithelial or submucosal. Whereas the layered separation derived from the clustering algorithm was a result of functional stratification, the manual annotation was used to filter out any ‘miss-classified’ spots. While there may be differences in the proximity of submucosal clusters to the epithelium between samples, this variability has been minimized in the differential gene expression analysis.

### 2.5 Deconvolution of spatial transcriptomic data

The SCDC package (29) was used to deconvolute the spatially resolved transcriptomic dataset at two separate levels of cellular granularity using a previously published single-cell (sc)RNA-seq dataset as a reference (30). The spatial data were filtered for spots containing <200 features and then sub-setted for a more equal distribution of spots per cluster, after which the top marker genes were selected for each cell type.

### 2.6 Differential gene expression and enrichment analysis

Differential gene expression analysis was performed to identify marker genes for each cluster, assess the differences between epithelial and submucosal spots, and determine differentially expressed genes (DEGs) across the two conditions (HIV^+^ vs. HIV**^−^**) using the Seurat function FindAllMarkers (test.use=wilcox). This analysis was conducted on a condensed version of the dataset, compiled by sampling a random selection of 25 spots for each epithelial cluster, and 50 spots for each submucosal cluster from each individual. Thereby, differences in biopsy size were accounted for, ensuring balanced representation across the dataset. Moreover, the clustering algorithm was designed to ensure that each cluster is composed of spots representing similar biologic entities, regardless of minor depth differences. The DEGs from each cluster were ordered based on log-fold change and subjected to Gene Set Enrichment Analysis (GSEA) (31) against the Gene Ontology (GO) database (32, 33). Genes and pathways with a false discovery rate (FDR) adjusted P-value <0.05 were considered significantly enriched.

### 2.7 Spatial trajectory analysis

We used the slingshot package (34) to evaluate the spatiotemporal dynamics of gene expression in each individual spot, which enabled the mapping of trajectory changes in gene expression for both epithelial and submucosal spots. Each spot was ordered based on the similarity in gene expression, allowing pseudo-measurement of distance throughout the tissue. This analysis takes the three-dimensional UMAP embedding and flattens it into a single dimension to show how gene expression patterns change across spots. Because spots in the same cluster have more similar gene expressions, they are closer together on this scale. The background color corresponding to the different clusters is added to highlight this.

### 2.8 Multi-epitope ligand cartography

Multi-epitope ligand cartography (MELC) was performed as previously described (35). Briefly, fresh frozen ectocervical tissue from the luteal phase was cut into 5-μm thick sections, placed on 3-aminopropyltriethoxysilane–coated cover slips (24 × 60 mm; Menzel-Gläser, Braunschweig, Germany), and fixed with 2% paraformaldehyde (Electron Microscopy Sciences, Hatfield, Philadelphia, USA). The samples were then blocked for 20 min with 10% goat serum and 1% bovine serum albumin in phosphate-buffered saline. A fluid chamber that holds 100 micro-liter was then attached to the slides using silicone sheets (Life Technologies, Carlsbad, CA, USA; 1.0 mm thickness), and multiplex histology was performed using a modified Toponome Image Cycler^®^ MM3 (MelTec GmbH & Co.KG Magdeburg, Germany) (36). Antibodies used for this assay are listed in **Supplementary Table S1** in the order in which they were applied during the cyclic MELC run. For each sample, two fields of view were acquired, measuring 666 × 666 μm each. Issues stemming from steric hindrance due to multiple antibodies were ruled out (37).

### 2.9 Image preprocessing

MELC is based on sequential staining of a single section, and thus, each acquired region must be registered using cross-correlation to a phase contrast image acquired at the beginning of the run. Background subtraction was performed by subtracting the previous bleaching image from subsequent fluorescence image to remove any residual signals from the previous image acquisition and autofluorescence. For each image, illumination correction was performed using a cubic-spline interpolation based on the minimum intensity values in the bleaching image from that fluorophore staining cycle to account for uneven illumination (38). The resulting 16-bit images were normalized in FIJI (v.2.9.0/1.53t) (39) using a rolling-ball algorithm, followed by removal of edges, and the image intensities were stretched to the full intensity range prior to analysis. The epithelial and submucosal compartments were manually annotated to ensure similar distribution across the study groups.

### 2.10 Cell segmentation

Images were uploaded to CellProfiler (v.4.2.1), and the intensities were adjusted based on a scale ranging from 0 to 1 (40). Initially, nuclei were identified based on 4’,6-diamidino-2-phenylindole (DAPI) staining and expanded to better reflect the size of the cells. Leukocytes were then identified based on CD45 staining with a threshold of 0.05. Lastly, the mean fluorescence intensity (MFI) of all markers was measured within each individual leukocyte and exported as a CSV file for further analysis (**Supplementary Table S1** **).**

### 2.11 Bioimage analysis

Bioimage analysis was performed in R (v.4.4.0) using several packages, including Seurat (v5.1.0), tidyseurat (v0.8.0), dplyr (v.1.1.4), and Harmony (v.1.2.0). The initial dataset containing leukocyte intensity values and metadata for all markers was imported as a CSV file. The expression data (i.e., intensity values) were then separated from the metadata, and a Seurat object of the expression data was generated; the metadata were subsequently integrated into this Seurat object to retain the contextual information for each cell. The expression data were normalized and scaled using the NormalizeData and ScaleData functions, respectively, in Seurat. We then identified variable features using FindVariableFeatures and performed PCA. The normalized data were also integrated in Harmony, and unsupervised clustering was performed using FindNeighbors and FindClusters to identify clusters representing distinct leukocyte populations. The data were then embedded into a 2D UMAP plot using the Seurat function RunUMAP. The identified clusters were further sub-clustered for a more detailed analysis of each immune cell population by repeating the same process outlined above. For everyone within the HIV^+^FSW and HIV^−^FSW groups, we calculated both the percentage and total counts of cells in each cluster to assess HIV-associated differences in leukocyte frequency and prevalence.

### 2.12 Statistical analysis

Fisher’s exact test was used to assess significance for all categorical variables of the clinical data, whereas the Mann–Whitney U test was used to evaluate significance for continuous clinical variables and bioimage analysis data. In all cases, we used a significance cut-off of a nominal P-value < 0.05. For differential expression analysis, genes and pathways with a false-discovery rate (FDR)-adjusted P-value < 0.05 were considered significant.

## 3 Results

### 3.1 Cohort description

Samples for this analysis were obtained from HIV^+^ and HIV^−^FSWs from the Pumwani Sex Worker Cohort, Kenya, who were recruited as a part of a larger longitudinal study (26). The sociodemographic and clinical characteristics for all cases are presented, including unadjusted p-values for each parameter (**Table 1**). In the current study, we aimed for a 1:1 ratio of HIV^+^ to HIV^−^ samples for both spatial transcriptomics and MELC, employing selection criteria that prioritized an RNA integrity number above 7 and a good tissue morphology. Of note, due to sample size limitations, the tissues analyzed by each methodology were collected at different points in the menstrual cycle. That is, for spatial transcriptomics, the samples were obtained during the follicular phase of the menstrual cycle, whereas the samples used for MELC were collected in the luteal phase. None of the women used hormonal contraceptives, as closely monitored prior to study inclusion, except for one participant in the MELC analysis who used DMPA (**Table 1**). This woman was therefore not considered to be in the luteal phase. In total, 10 follicular phase ectocervical biopsies were analyzed by spatial transcriptomics (HIV^+^FSWs, n = 5; HIV^−^FSWs, n = 5). Luteal phase biopsies from the same 10 women were analyzed by MELC, along with luteal phase samples from an additional six women (n = 16 total) to account for greater variability in imaging data (HIV^+^FSWs, n = 8; HIV^−^FSWs, n = 8). In all cases, menstrual phases were defined based on self-reported time since last menses and further validated by measuring plasma levels of estradiol and progesterone.

**Table 1:** Characteristics of cohort at time of sample collection.

Variables	Spatial transcriptomics			MELC		
	HIV^+^ FSWs n=5 Median (range or %)	HIV^-^ FSWs n=5 Median (range or %)	P-value	HIV^+^ FSWs n=8 Median (range or %)	HIV^-^ FSWs n=8 Median (range or %)	P-value
**Age** (years)	40 (35-45)	36 (30-40)	0.15^1^	40 (35-48)	36 (30-40)	0.03^1^
**Time in sex** work^a^ (months)	102 (36-156)	108(24-120)	0.95^1^	102 (36-156)	108 (24-120)	0.99^2^
Not available	1 (20%)	0	0.99^2^	2 (25%)	0	0.48^2^
**Time since diagnosis**^**b**^ (months)	51 (31-63)	NA		48 (27-63)	NA	NA
Not available	1 (20%)			2 (25%)		
**Regular Partner**^**c**^						
Yes	4 (80%)	3 (60%)	0.99^2^	5 (63%)	4 (50%)	0.61^2^
Not available	0	0		1 (13%)	0	0.99^2^
**DMPA use**^**d**^						
Yes	0	0		1 (13%)	0	0.47^2^
Not available	0	0		1 (13%)	0	0.99^2^
**Days since menses**^**e**^	11 (8-22)	13 (4-14)	0.94^2^	22 (8-28)	25 (16-25)	0.46^1^
Not available	0	0		2 (25%)	0	0.47^2^
**Estradiol levels** Plasma; pg/ml	57 (24-206)	78 (22-242)	0.75^1^	250 (40-510)	125 (50-620)	0.98^1^
Not available	0	0		1 (13%)	0	0.99^2^
**Progesterone levels** Plasma; ng/ml	0.2 (0.05-8.5)	0.1(0.05-1.9)	0.69^1^	3.3 (0.9-15.9)	6.3 (0.2-11.3)	0.49^1^
Not available	0	0		2	0	0.47^2^
**Bacterial Vaginosis**^**f**^			0.29^2^			0.26^2^
BV	2 (40%)	1 (20%)	0.99^2^	1 (13%)	1 (13%)	0.99^2^
Intermediate	2 (40%)	0 (0%)	0.44^2^	0	3 (38%)	0.20^2^
Normal	1 (20%)	4 (80%)	0.21^2^	6 (75%)	4 (50%)	0.28^2^
Not available	0	0	NA	1 (13%)	0	0.99^2^
**Cervicovaginal microbiome**^**g**^						
L1	0	0		0	0	
L2	0	3 (60%)	0.17^2^	1 (13%)	4 (50%)	0.30^2^
L3	1 (20%)	1 (20%)	0.99^2^	2 (25%)	2 (25%)	0.99^2^
L4	3 (60%)	1 (20%)	0.21^2^	3 (38%)	2 (25%)	0.58^2^
L5	0	0		0	0	
Not available	1 (20%)	0	0.99^2^	2 (25%)	0	0.47^2^
**STI screening**^**h**^						
No	5 (100%)	5 (100%)		8 (100%)	8 (100%)	
**CD4 cell count** (cells/ml)	590 (505-667)	1,040 (749-1,180)	0.008^1^	570 (442-961)	901 (476-1,508)	0.15^1^
Not available	0	0		1 (13%)	0	0.99^2^
**HIV RNA** (copies/ml)	672 (20-77,600)	NA	NA	1824 (20-77,600)	NA	NA
Not available	0			1 (13%)		

a Self-reported time in sex work.

b If already HIV seropositive at time of inclusion in the Pumwani sex worker cohort: Time since inclusion in the cohort.

c Regular partner indicates having a regular partner in addition to other sex work clients (self-reported).

d DMPA: Regular use (since more than 6 months) of depot medroxyprogesterone acetate. The non-DMPA users did not use any type of hormonal contraceptives.

e Days since the first day of the most recent menstrual period. Participants using DMPA are not included.

f Bacterial vaginosis (BV) as defined by Nugent Score (BV: 7-10; Intermediate: 4-6; Normal: 0-3).

g Cervicovaginal luminal microbiome during the follicular phase (16S rRNA), as previously defined (23) as well as obtained from the Gene Expression Omnibus (GSE217237). L1; > 80% abundance of *L. crispatus* and/or *L. Jensenii*. L2; > 80% *L.* spp. L3; > 10% *Gardnerella vaginalis* and/or < 5% *Prevotella*. L4; > 5% *Prevotella*. L5; Samples that did not fit within L1-L4.

h STI screening at time of sample collection: Diagnosis of any of the sexually transmitted infections *Chlamydia Trachomatis*, *Neisseria Gonorrhoeae*, *Treponema Pallidum* and *Trichomonas Vaginalis* was an exclusion criterium at study enrolment. These diagnostic tests were repeated at time of sample collection for the present sub-study. No=negative screening result.

FSW, female sex worker; NA, Not applicable; DMPA, Depot medroxyprogesterone acetate; BV, Bacterial vaginosis.

P-values: ^1^Mann Whitney U test; ^2^Fisher’s exact test.

For samples undergoing spatial transcriptomics, the participant ages were comparable between the HIV^+^FSW and HIV^−^FSW groups; however, the average age of HIV^+^FSWs was significantly higher than that of HIV^−^FSW when including all participants who provided samples for MELC (P = 0.03). Time spent in sex work and the prevalence of bacterial vaginosis, as assessed by Nugent’s score, were also comparable between the two study groups. Furthermore, we detected no differences in the cervicovaginal microbiome composition of HIV^+^ and HIV^−^FSWs as assessed by molecular 16S ribosomal RNA-sequencing. All participants were negative for *Chlamydia trachomatis, Neisseria gonorrhoeae, Treponema pallidum*, and *Trichomonas vaginalis* infection per study enrollment criteria. The average number of CD4^+^ cells/ml was significantly decreased in HIV^+^ vs. HIV^−^ FSWs for samples undergoing spatial transcriptomics (P = 0.008), although no significant differences were observed between the study groups for samples undergoing MELC. Plasma viral load was assessed at the follicular phase of the menstrual cycle for all HIV^+^FSWs, and none of the study participants were on antiretroviral therapy.

### 3.2 Characterization and clustering of gene expression patterns within the ectocervical mucosa across samples

In-depth transcriptional studies with detailed spatial resolution within the FRT during chronic HIV are lacking. We, therefore, assessed the gene expression patterns within ectocervical biopsies from HIV^+^ and HIV^−^FSWs using spatial transcriptomics, which allows for the visualization and analysis of molecular changes occurring between groups while still preserving the spatial information in each tissue. For these analyses, we assessed mRNA expression within each spot measuring 55 μm in diameter, which represents between 1–10 cells. The resulting dataset from all samples comprised 13,091 spots, with a median of 3,341 transcripts and 1,691 genes per spot.

**Figure 1:**
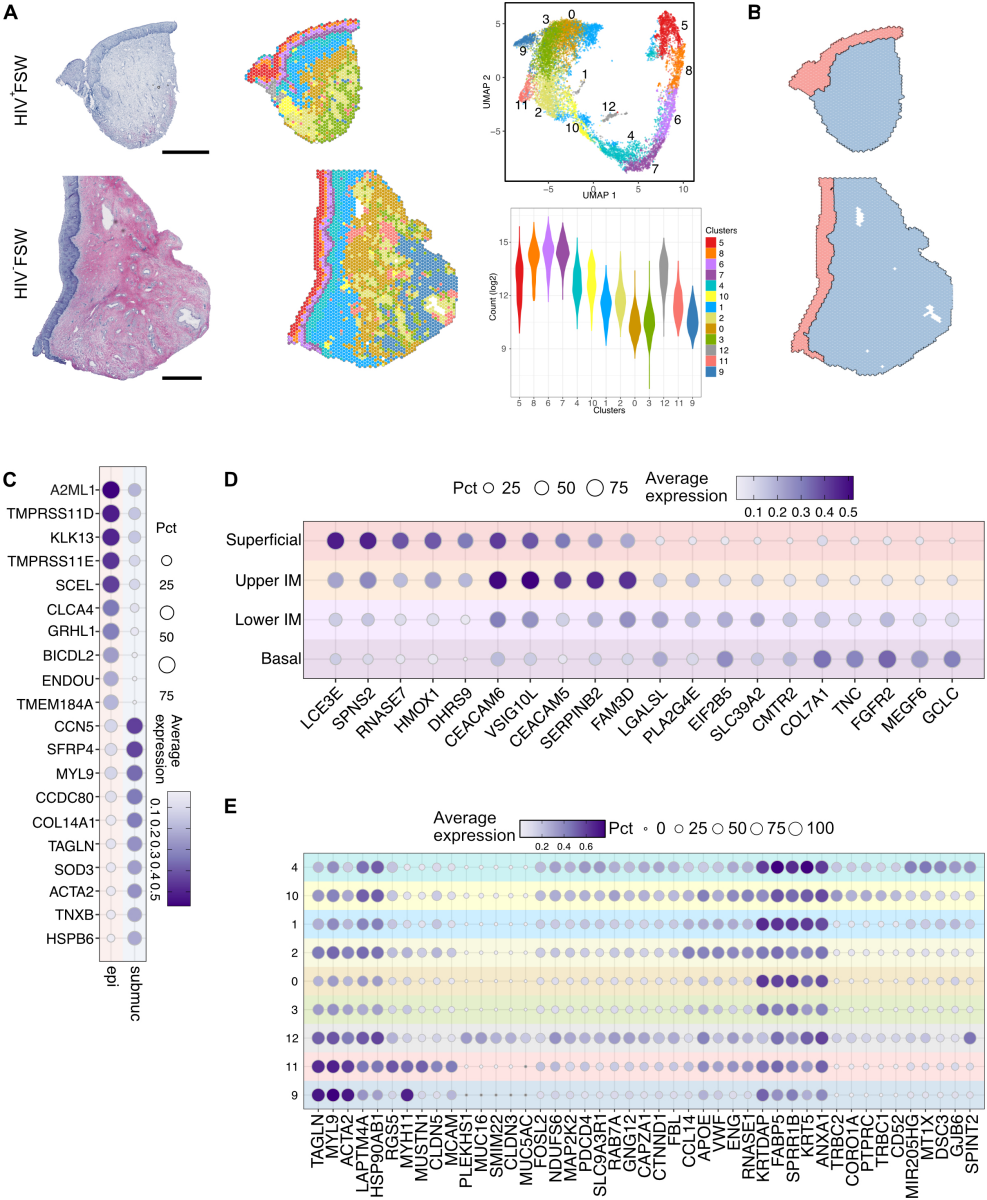
Overview of the ectocervical transcriptional profiles within the epithelial and submucosal compartments. **(A)** Examples images of H&E staining of ectocervical tissue samples from the HIV^+^FSWs (upper) and the HIV^−^FSWs (lower) and unsupervised clustering determined by UMAP analysis of gene expression profiles plotted on ectocervical tissue (Pat-ID P023 and P067). A total of 13 clusters were identified (four epithelial and nine submucosal); the RNA count in each cluster is listed as an indication of cell density (lower right). **(B)** Manual classification of ectocervical tissue into epithelial and submucosal regions based on morphology to assess the gene expression profile within each compartment. **(C)** The top genes differentially expressed in the manually annotated epithelial vs. submucosal regions. **(D, E)** The top five individual marker genes expressed in each of the **(D)** four epithelial clusters and **(E)** nine submucosal clusters compared to all other clusters are shown. Scale bars represent 0.5 mm. H&E, hematoxylin and eosin; FSW, female sex worker; UMAP, uniform manifold approximation and projection.

We then performed unsupervised clustering based on gene expression profiles, assigning each spot to a specific cluster (41). In total, 13 clusters were identified across all samples, four of which (clusters 5–8) were defined as epithelial, and the remaining nine (clusters 0–4 and 9–12) were defined as submucosal (**Figure 1A**). The epithelial clusters showed the highest mRNA expression levels, which may reflect the greater density and proliferative capacity of epithelial cells relative to submucosal cells (42).

Next, we manually annotated the epithelial and submucosal tissue based on morphology to assess the gene expression profile within each compartment (**Figure 1B**). Differential gene expression analysis between the two compartments revealed 9,957 DEGs, of which 3,960 were upregulated and 5,997 were downregulated with FDR-adjusted P < 0.05. As expected, epithelial tissue showed upregulation of keratinocyte markers (*A2ML1, SCEL* and *GRHL1*), whereas gene expression within the submucosa was more associated with fibroblasts (*COL14A1* and *CCN5*) and smooth muscle cells (*ACTA2*) (**Figure 1C**; **Supplementary Table S2**). Of note, the submucosa also contains blood vessels which contribute to the expression of associated gene markers.

The epithelium was further separated into four distinct layers: a superficial (cluster 5), an upper intermediate (cluster 8), a lower intermediate (cluster 6), and a basal (cluster 7) layer (**Figure 1A**; **Supplementary Figure S1**). When assessed across tissue samples representing both study groups, the superficial and upper intermediate layers displayed distinct transcriptional profiles. Specifically, the superficial layer was predominantly characterized by expression of genes associated with the terminal differentiation of epithelial cells (*LCE3D/E* and *SCEL*) and antimicrobial activity (*RNASE7*, *ERO1A*, and *LCN2*), potentially due to its proximity to the cervical lumen. In contrast, the upper intermediate layer showed elevated expression of genes involved in epithelial adhesion and differentiation (*CEACAM5/6, DSG3*, and *FAM3D*) and immunoregulation (*ANXA1*, *CD24*, and *ATP1B1*). The lower intermediate and basal layers expressed similar marker genes and were not as distinctly separated as the upper two layers. Distinct markers expressed in the lower intermediate layer included those involved in epithelial tissue repair and stability (*PKP1*, *KRT19*, *FGFBP1*) and cellular metabolism (*HMGCS1*, *LPIN 1, FABP5*), whereas the basal layer showed expression of genes related to epithelial anchoring (*COL7A1*) and cell proliferation (*FGFR2*) (**Figure 1D**; **Supplementary Table S2**).

In contrast to the epithelial clusters and submucosal clusters 4 and 1 (near the basal membrane), the deepest submucosal clusters exhibited a less structured distribution across samples (**Figure 1A**; **Supplementary Figure S1**). Moreover, the gene expression patterns across submucosal clusters were more like one another than those of the epithelial clusters based on the top marker genes in each cluster (**Figure 1E**). Among the submucosal clusters, cluster 4 exhibited enrichment for the expression of genes linked to epithelial stability and adhesion to the underlying extracellular matrix, likely due to some spots covering both the epithelial and submucosal tissue regions. In contrast, cluster 10 showed a significant upregulation of genes associated with leukocytes (*PTPRC*) and T cells, particularly, marked by *TCRB1* and *TCRB2*. Cluster 1 expression profiles were largely associated with inflammation and immune responses, featuring the expression of genes such as *ANXA1* and *FABP5*, whereas cluster 2 was characterized by the expression of endothelial marker genes, including *ENG*, *VWF*, and *CCL14*. Of note, cluster 12 was observed in only two HIV^+^FSWs and was, therefore, not explored further in this study. Lastly, clusters 9 and 11 were found to predominantly express genes associated with smooth muscle cells (**Figure 1E**).

### 3.3 Increased innate and adaptive immune activation within the cervical epithelium associated with HIV infection

**Figure 2:**
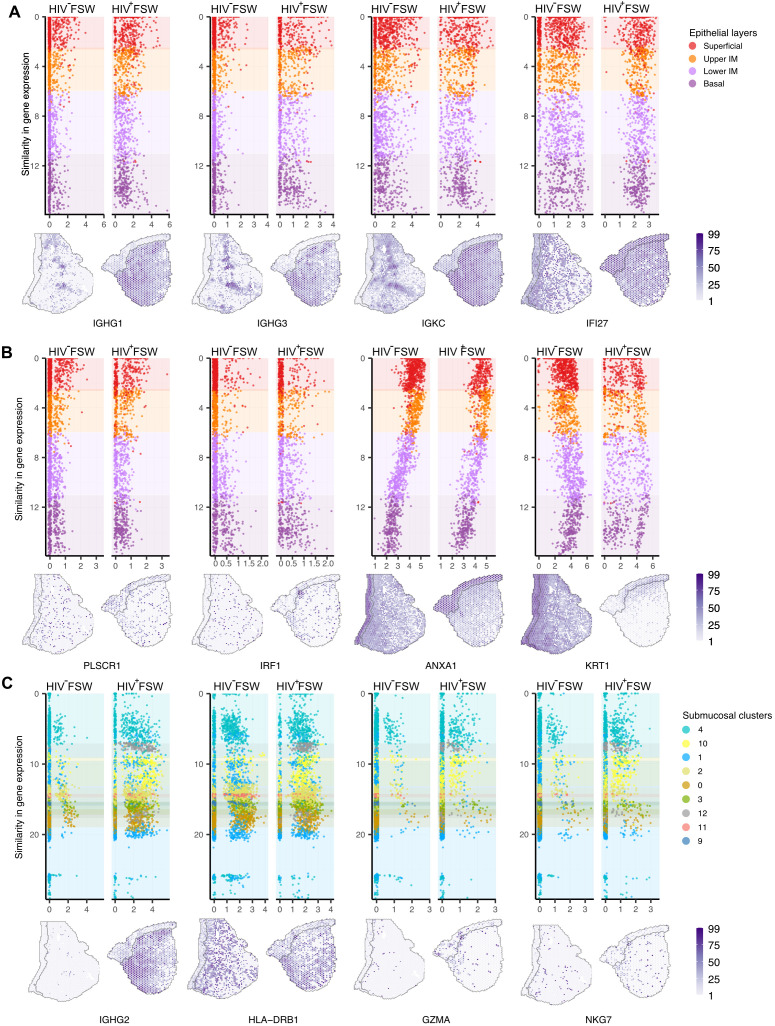
Differential ectocervical gene expressions associated with chronic HIV infection. The spatiotemporal dynamics of gene expression is here demonstrated at the individual spot level. Spots are ordered by similarity in gene expression, resulting in a pseudo measurement of distance across the mucosa. The color codes represent different clusters. **(A)** Selected upregulated genes within all four epithelial layers (Y-axis) in HIV^+^FSW and HIV^−^FSW plotted relative to the expression (X-axis) of selected genes with gene expression values plotted on tissue below. **(B)** Selected upregulated genes within the superficial layer (*PLSCR1*), upper intermediate (*IRF1*) and the lower intermediate and basal layer (*ANXA1*) as well as across all epithelial layers the *KRT1* gene was downregulated, comparing HIV^+^FSWs with HIV^−^FSWs, with gene expression values plotted on tissue below. **(C)** Selected upregulated genes across cluster 4, 10 and 1 (*IGHG2*), cluster 4 and 1 (*HLA-DRB1*) and cluster 4 (*GZMA* and *NKG7*) in HIV^+^ vs. HIV^−^FSWs, with gene expression plotted on tissue below. Plotted individuals: Pat-ID P023 and P067. FSW, female sex worker; DEG, differentially expressed gene. IM, intermediate layer.

We next performed a thorough assessment of the transcriptional differences within ectocervical tissues from each group, comparing profiles observed in HIV^+^FSWs to those detected in HIV^−^FSWs. Across the epithelium, we identified 299 DEGs, of which 174 were upregulated and 125 were downregulated with FDR-adjusted P < 0.05 in the HIV^+^FSWs. Among the upregulated genes, most were immunomodulatory, including those involved in antimicrobial activity mediated by innate (*IFI27*, *DEFB4A*, *PI3*, and *S100A2/4/6/7*) and adaptive (*ANXA1* and *IGHG1/2/3/4*) immunity. In addition to *IGHG* genes, an upregulation of light chain related genes including *IGKC* and *IGLC1* also demonstrated an epithelial wide induction highlighting its physiological relevance (**Figure 2A**; **Supplementary Table S3**). Therefore, to better understand how each layer was associated with chronic HIV infection, we identified the unique DEGs between study groups within each layer (**Supplementary Table S4**). The superficial layer contained the lowest numbers of unique DEGs, most of which were predicted to be involved in inflammation (*TFF3* and *PLSCR1)* and antimicrobial activity (*C3* and *PIGR*), whereas the upper intermediate layer predominantly included DEGs associated with IFN-induced (*IRF1*, *IFI16/44* and *ISG15*) antiviral defenses (*APOBEC3A* and *BST2*). Although innate immunity genes were also expressed within the lower intermediate and basal layer, these regions also showed upregulation of genes involved in cellular immunity (*ANXA1, CD82, GZMA/K*, and *KRT17*). Interestingly, genes involved in humoral immune responses and some IFN-induced genes (*IFI27*) were prominently expressed across all layers (**Figure 2B**; **Supplementary Table S4**).

Downregulated genes within the epithelium in the HIV^+^ vs. HIV^−^ group were predicted to be primarily associated with epithelial integrity (*LOR, FLG*, and *KRT 1/4/10/13*), metabolism (*ALOX12, PLA2G4D, RXRA*, and *CRABP2*), and proliferation (*SERPINB13* and *FOSL2*). Moreover, the superficial and upper intermediate layers showed an HIV-associated downregulation of genes important for terminal differentiation of keratinocytes (*SPRR3*, *LOR*, and *FLG*). Most downregulated genes were observed within the lower intermediate and basal layers and represent a broad array of molecular functions, including epithelial integrity (*KRT 1/4/10/13*), desmosomal structure (*DSG1, DSC2*, and *PKP1*), and metabolism (*RXRA*, *ALOX12*, and *CRABP2*) (**Figure 2B**; **Supplementary Table S4**).

### 3.4 HIV infection is associated with submucosal immune activation characterized by humoral and cellular immune responses

Across all submucosal clusters, 310 DEGs were identified, of which 220 were upregulated and 90 downregulated when comparing the two study groups. Of note, abundant upregulation of the humoral immune response, as indicated by the expression of several antibody isoforms, was evident throughout the submucosal clusters in HIV^+^FSWs (*IGHG1/2/3, IGLC1/2/3*, and *IGHM*) (**Supplementary Table S3**). In addition to the humoral immune response, we further observed an upregulation of cellular immunity in multiple submucosal clusters, including *HLADRB1/DRB5, CD3D, TRBC2, CCL5, IFI27*, and *IFITM1* (**Supplementary Table S3**). Several genes downregulated within the submucosa of HIV^+^FSWs were predicted to be involved in cellular metabolism (*ALOX12* and *CRABP2*) and tissue structure (*SPRR3, KRT1/4, COL1A2* and *COL14A1*) (**Supplementary Table S4**).

Given that most immune cells within the cervical mucosa are aggregated around the basal membrane of the epithelium, we focused on clusters 4 and 1 due to their proximity to the basal membrane, as well as cluster 10 based on its immunological characteristics. Notably, for cluster 10, a higher number of immune cell genes in a spot can indicate a higher number of immune cells, but this correlation is not always direct. Cluster 4, located adjacent to the basal membrane, expressed the largest set of unique DEGs in HIV^+^FSWs, exhibiting upregulation of several genes encoding ribosomal proteins, which indicates an increase in translational activity (**Supplementary Table S4**). Notably, several human leukocyte antigen (HLA)-related genes were also upregulated within cluster 4 (*HLA-A/DPB1/DQB1/DQA1/DRA*), signifying an increase in antigen presentation and immune activation in response to HIV infection. In addition, we observed the upregulation of several genes involved in antiviral activity and cellular immunity in cluster 4, including marker genes for both T cells and natural killer (NK) cells (*NKG7*, *IRF1, CD48, HCST, GZMA*, and *CCL4*) (**Figure 2C**; **Supplementary Table S4**). Consistent with the increase in antibody-encoding genes observed throughout all submucosal clusters, cluster 4 further showed elevated expression of several additional humoral genes (*C1QB* and *C1QC*) in the HIV^+^FSW group. Cluster 1 contained fewer unique DEGs in HIV^+^FSWs than cluster 4. However, increased expression of *TRBC1* and *TRBC2* was observed within this cluster in tissue from HIV^+^FSWs, indicating enhanced proliferation or recruitment of T cells. Similarly, cluster 10 exhibited elevated expression of general pro-inflammatory markers and genes related to B cell–mediated immune responses (*CD81, MZB1*, and *C3*) in HIV^+^ vs. HIV^−^FSWs. Collectively, these results are indicative of HIV-associated cervical immune activation characterized by IFN signaling and adaptive immune responses encompassing both humoral and cellular responses.

### 3.5 GSEA reveals an HIV-associated alteration of immunoregulatory and developmental-related pathways in the ectocervix

**Figure 3:**
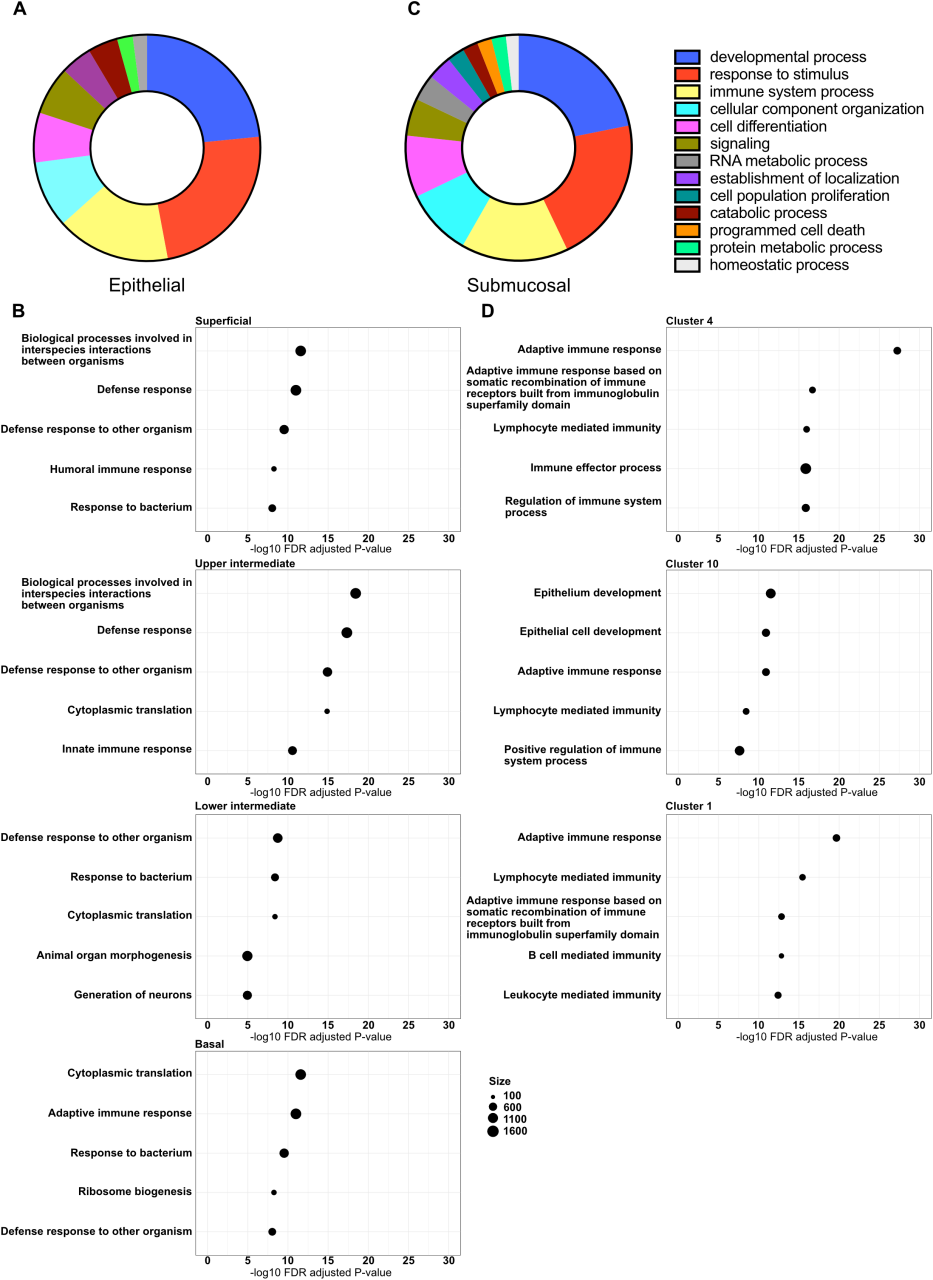
Altered major biological pathways associated with chronic HIV infection. Gene expression profiles within all clusters identified by spatial transcriptomics were subjected to GSEA using the GO database. **(A)** The GO terms enriched in HIV^+^FSW vs. HIV^-^FSWs were summarized using GO Slim to identify the major HIV-associated altered biological pathways within the epithelium, and **(B)** the top 5 enriched GO terms (partly functionally overlapping) of the four epithelial layers. **(C)** The summarized GO slim terms of the submucosa, and **(D)** the top 5 enriched GO terms (partly functionally overlapping) of three submucosal clusters 4, 10 and 1. Figure A and C depict the percentage of pathways that were categorized within each higher-level pathway. GO, Gene Ontology; GSEA, Gene Set Enrichment Analysis; FSW, female sex worker.

To determine functional differences and identify biological pathways altered in response to chronic HIV infection within the different tissue compartments and cell clusters, we performed GSEA using the GO database (32, 33). The identified GO pathways were then summarized using GO Slim to identify the most prominent biological processes altered within the different compartments. Within epithelium, we identified 17 major altered biological pathways in tissue from HIV^+^FSWs, representing a broad array of molecular functions. However, three of these pathways stood out, including response to stimulus (116 pathways), developmental processes (115 pathways), and immune system processes (80 pathways) (**Figure 3A**). The developmental processes group largely comprised pathways involving epithelial maintenance, thus reflecting the downregulation of several key genes involved in epithelial stability, whereas enrichment for pathways associated with response to stimuli and immune system processes is reflective of the upregulation of genes with immunomodulatory functions. Additionally, we identified several pathways involved in different metabolic processes (**Figure 3A**; **Supplementary Table S5**).

We further identified GO pathways differentially altered between the two study groups within each epithelial layer. Within tissue from HIV^+^FSWs, the top enriched pathways were similar in all layers, primarily involving antimicrobial defense; innate, humoral, and cellular immune responses; and metabolic processes. The superficial, upper, and lower intermediate layers also had similar numbers of differentially altered biological pathways (141, 162, and 140 pathways, respectively), whereas twice as many were identified in the basal layer (296 pathways). Additionally, the top-identified pathways enriched within the basal layer included a larger number of metabolic and cellular organization pathways than those in other layers, likely reflecting increased proliferation within the basal layer (**Figure 3B**; **Supplementary Table S5**).

GO slim analysis of the whole submucosal compartment, including all submucosal clusters, revealed 18 major biological pathways differentially altered between the two study groups. Similar to the epithelium, the three major pathways enriched in the submucosa from HIV^+^FSWs were developmental processes, response to stimulus, and immune system processes (168, 162, and 118 pathways, respectively), with cell differentiation (68 pathways), signaling (41 pathways), and different metabolic processes enriched to a lesser extent (**Figure 3C**; **Supplementary Table S5**). Next, we assessed the GO pathways within clusters 4, 10, and 1 that were differentially enriched in tissue from HIV^+^ vs. HIV^−^FSWs. Intriguingly, more altered pathways were detected in these clusters (257, 246, and 257 pathways, respectively) than were observed in the epithelial layers, excluding the basal layer. All showed significant alterations within immunological processes, primarily through both cellular and humoral adaptive immunity. Moreover, the top two altered pathways within the immunological cluster 10 were identified as the general term “epithelium development” and the more specific term “epithelial cell differentiation,” which could reflect an increased density of immune cells closer to the basal membrane of the epithelium within the ectocervix of women living with HIV relative to those without HIV (**Figure 3D**; **Supplementary Table S5**).

### 3.6 Spatial mapping of gene expression indicates no HIV-associated differences in ectocervical cell populations

**Figure 4:**
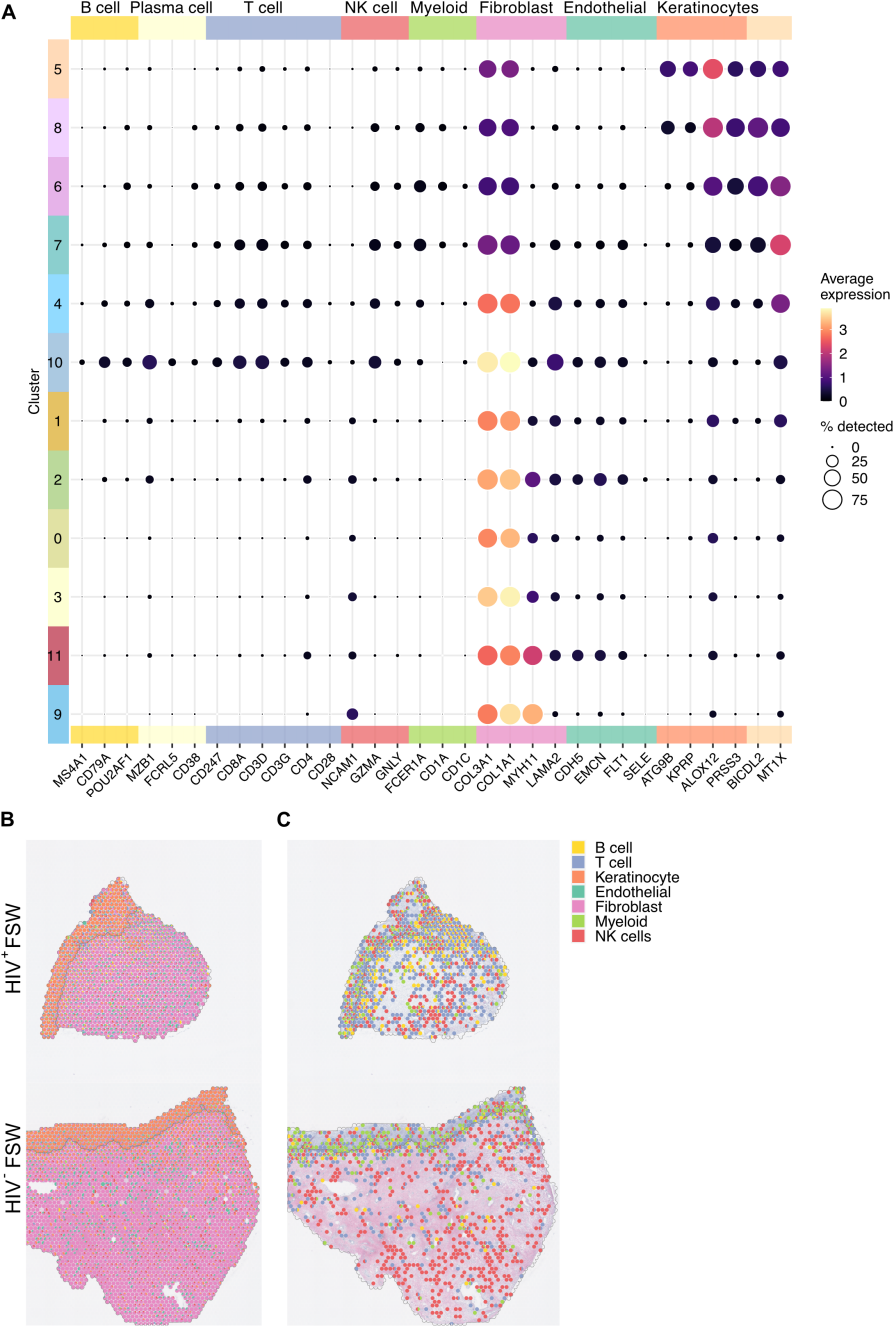
Visualization of the cell populations present within the ectocervix. **(A)** The average gene expression levels of cell markers for the distinct cell populations present within the clusters identified in the cervix. **(B)** Relative gene expression levels of the select cell markers in each spot illustrated as a pie chart plotted on tissue (Pat-ID P023 and P067). **(C)** Given the predominance of keratinocytes and fibroblasts across the analyzed tissue, keratinocyte- and fibroblast-related genes were excluded to better visualize immune cell distribution in tissue of HIV^+^FSWs and HIV^−^FSWs. FSW, female sex worker.

We next used a scRNA-seq dataset (30) to deconvolute the cellular composition of the ectocervical mucosa and determine the proportional cellular composition within each spot. Results show that nearly all epithelial spots expressed markers of keratinocytes, 91% of which were classified as epithelial cells. The submucosal tissue displayed a greater heterogeneity but was largely classified as fibroblasts (72%) (**Figure 4**). T and NK cell–related genes were most prevalent within the epithelial clusters and in clusters 4 and 10. The highest expression of B cell–related genes were seen within cluster 10 (**Figure 4A**). Throughout the tissue, 0.4%, 0.3%, and 3.5% of all spots were classified as T cells, B cells, and other immune cells, respectively (**Figure 4B**; **Supplementary Table S6**). Of note, despite the observed upregulation of both cellular and humoral immunity in tissue from HIV^+^FSWs, no significant differences in cell composition could be observed between the two study groups (**Supplementary Table S6**). Therefore, to further investigate the immune-specific cell populations within the ectocervix, we excluded the epithelial and fibroblast genes from our analyses, given that keratinocyte- and fibroblast-related genes were overrepresented in these tissues. The most prevalent immune cell population identified with this strategy was largely aggregated around the basal membrane of the epithelium (**Figure 4C**), which confirms the legitimacy of our unsupervised clustering approach. In addition, visual examination of the immune cell population within the ectocervix revealed an increased leukocyte prevalence in tissue from HIV^+^FSWs (**Supplementary Figure S2**).

### 3.7 HIV infection is associated with increased leukocyte density primarily driven by CD8^+^ T cells

To assess the immune cell populations, present in the ectocervix in response to chronic HIV infection in more detail, we next used the advanced imaging technique known as MELC. This method is based on the repeated staining, imaging, and bleaching of tissue sections, which allows for simultaneous visualization of multiple biomarkers within a sample, thus providing a comprehensive analysis of immune cell populations and tissue architecture. Ectocervical tissue biopsies from HIV^+^ (n = 8) and HIV^−^FSWs (n = 8) were subjected to nuclear staining (DAPI) and analyzed using a pre-defined panel of 17 immune cell markers, which were selected to identify distinct T cell subsets (**Supplementary Figure S3**; **Supplementary Table S1**). Two fields of view, each covering an area of 0.44 mm^2^ and containing both epithelial and submucosal tissue, were acquired per individual tissue, generating a stack of 18 images per field of view. The epithelial and submucosal compartments were manually annotated to ensure a similiar tissue distribution between the study groups. While the total area was pre-defined for all samples, the epithelial and submucosal areas were also found comparable between the study groups (**Supplementary Figure S3**).

**Figure 5:**
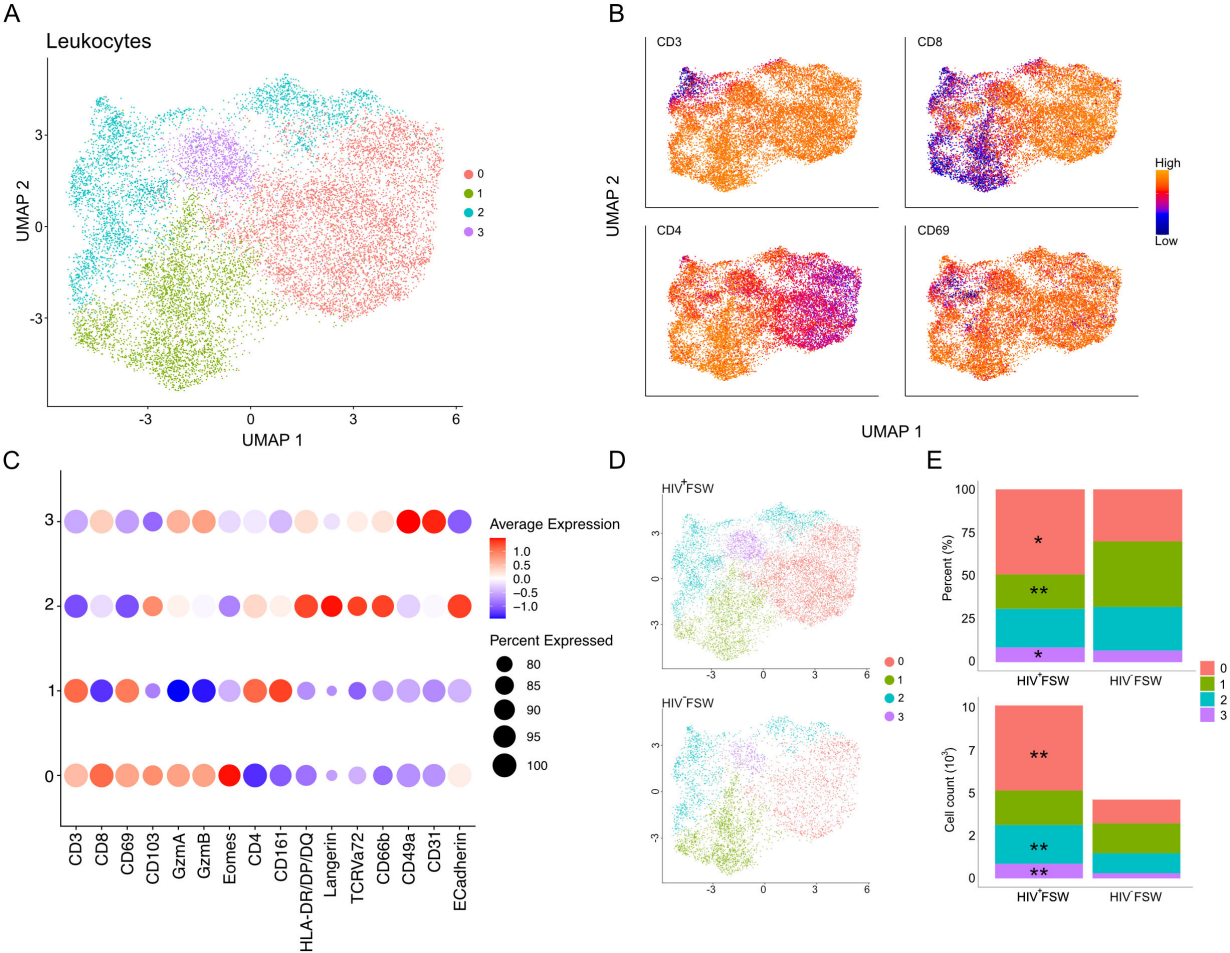
Altered cervical immune cell populations are present in women with chronic HIV infection. **(A)** UMAP analysis followed by unsupervised clustering of ectocervical leukocytes from MELC-stained tissue sections. **(B)** Feature plot visualizing cellular protein expression levels of CD3, CD8, CD4, and CD69. **(C)** Dot plot visualizing MFI levels of individual markers within each cluster. **(D)** UMAP plot showing the clustering and leukocyte prevalence within the HIV^+^FSWs and HIV^−^FSWs. **(E)** Bar chart showing the frequencies (upper) and total counts (lower) of cells within each cluster in the two study groups. Statistical significance was determined using the Mann–Whitney U test with significance set at P < 0.05. *, P < 0.05; **, P < 0.01. FSW, female sex worker; MELC, multi-epitope ligand cartography; MFI, mean fluorescence intensity; UMAP, uniform manifold approximation and projection.

Cells were identified based on DAPI staining, and leukocytes were subsequently identified based on CD45 expression. The cell density (cells/mm^2^) was similar between the two study groups. However, a significantly increased density of CD45^+^ leukocytes was observed in ectocervical tissue from HIV^+^ FSWs (**Supplementary Figure S3**). We then measured the MFI of staining for all markers present within leukocytes and performed UMAP to assess the ectocervical immune cell population in an unbiased manner. Results showed that the leukocytes were distributed into four clusters based on similarities in expression of all markers (**Figure 5A**). Clusters 0 and 1 exhibited high expression of CD3 and were classified as T cells. Cluster 0 expressed elevated levels of CD8, and cluster 1 expressed high levels of CD4; thus, these clusters were classified as CD8^+^ and CD4^+^ T cells, respectively. Both T cell clusters showed high expression of CD69, indicating a significant presence of TRM T cell phenotypes although we recognize that additional markers would be needed to fully confirm these cells as TRMs. In contrast, clusters 2 and 3 displayed low or no expression of CD3 and were considered a mix of non-T cells and CD3^low^ T cells. Cluster 2 showed elevated HLA-DR/DP/DQ, Langerin, and CD66b expression, along with moderate expression of CD8 and CD4, suggesting it included several mucosal immune cell populations, such as NK cells, macrophages, and Langerhans cells. Cluster 3 was predominantly identified based on elevated co-expression of CD49a and CD31 (**Figures 5B, C**). However, we are aware that due to the need for cell segmentation, there are cross-contamination issues for adjacent and tiled cells affecting the expression profiles of the cells.

Next, we assessed the association between chronic HIV infection and each cell population. Given that the HIV^+^FSWs had a 2.2-fold increase in the number of leukocytes identified by MELC compared to HIV^−^FSWs (10,155 vs. 4,631), we measured both the frequencies and total counts of cells comprising each cluster in every participant (**Figures 5D, E**; **Supplementary Figure S3**). Analysis of cluster frequency revealed a significant relative increase in CD8^+^ T cells (cluster 0) within tissue from HIV^+^FSWs. In parallel, we detected a significant relative decrease in CD4^+^ T cells (cluster 1) within the HIV^+^ group. While no difference in frequency could be observed for cluster 2, there was a slight increase in cluster 3. Consistent with these findings, we detected a significant increase in CD8^+^ T cell count within HIV^+^FSWs relative to HIV^−^FSWs. However, no differences in CD4^+^ T cell counts could be observed between the two study groups. Additionally, we observed a significantly increased number of cells in cluster 2, characterized by non-T cells and CD3^low^ T cells, and cluster 3 characterized by CD31, CD49a expression, in the HIV^+^FSW group (**Figure 5E**; **Supplementary Table S7**).

Collectively, these results highlight the association of chronic HIV infection on the cervical immune populations, revealing a significant increase in leukocytes, primarily mediated by an increase of CD8^+^ T cells, in women living with HIV. Moreover, although a proportional decrease in CD4^+^ T cells was observed within the HIV^+^FSWs, we detected no differences in the total number of CD4^+^ T cells present within the cervix of these individuals.

### 3.8 CD8^+^ TRM cells comprise the predominant cervical leukocyte population and are expanded in HIV^+^FSWs

We further characterized the MELC-identified T cell populations in ectocervical tissue of our study subjects by analyzing each cluster individually. The epithelial and submucosal compartments were here defined by E-cadherin expression, which is an abundantly expressed junctional protein covering approximately 70-80% of the ectocervical epithelium (43). Thus, clusters demonstrating high expression of E-cadherin were defined as epithelial, while E-cadherin-negative clusters were defined as submucosal.

**Figure 6:**
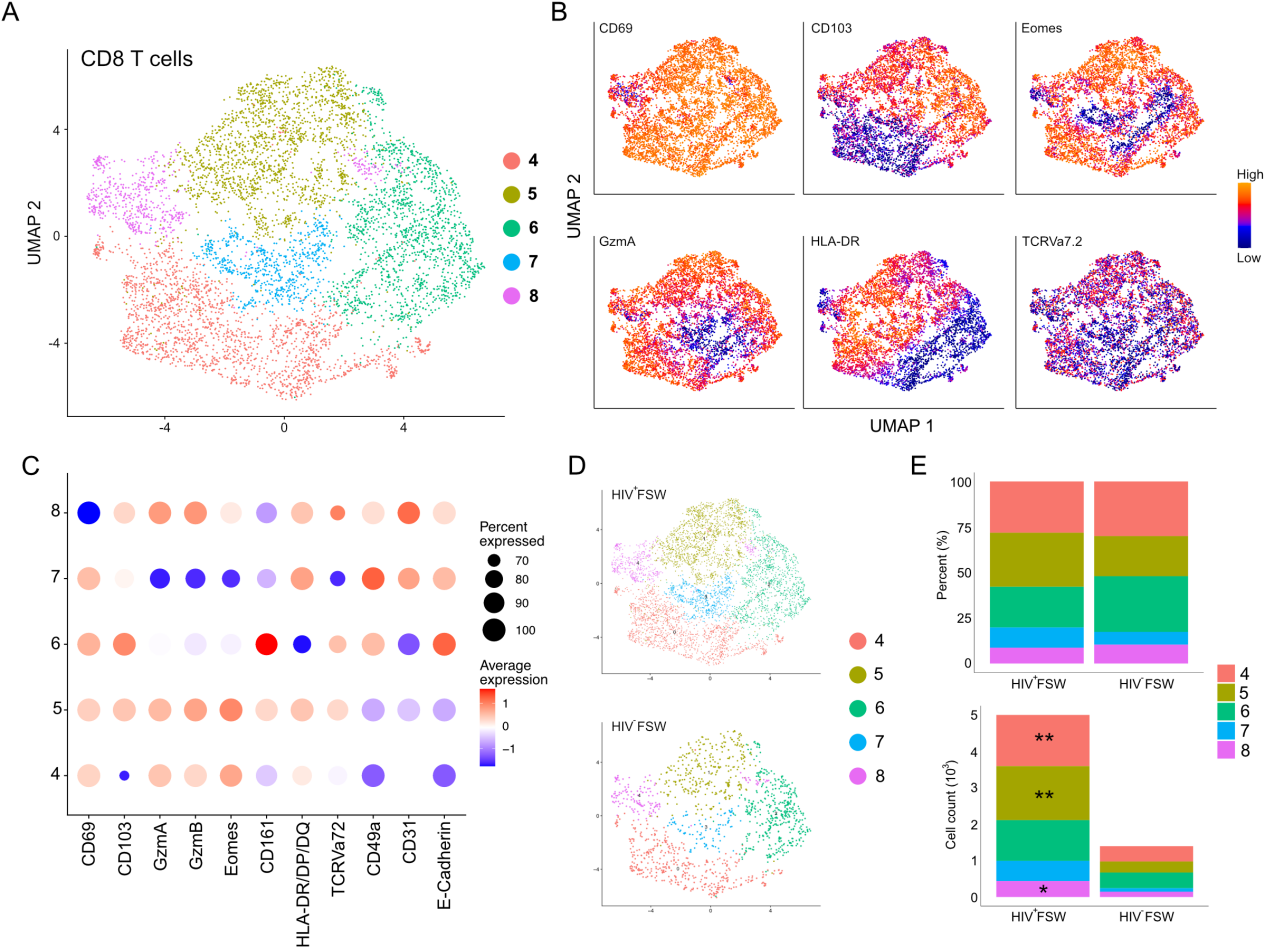
Increased cervical CD8^+^ T cells are observed in women with chronic HIV infection. **(A)** UMAP analysis followed by unsupervised clustering of ectocervical CD8^+^ T cells from MELC-stained tissue sections. **(B)** Feature plot visualizing cellular protein expression of CD69, CD103, Eomes, GzmA, HLA-DR/DP/DQ, and TCR Vα7.2. **(C)** Dot plot visualizing MFI levels of individual markers within each CD8^+^ T cell sub-cluster. **(D)** UMAP plot showing the sub-clustering and CD8^+^ T cell prevalence within HIV^+^FSWs and HIV^−^FSWs. **(E)** Boxplots showing the frequencies (upper) and total cell counts (lower) of the CD8^+^ T cell clusters within the two study groups. Statistical significance was determined using the Mann–Whitney U test with significance set at P < 0.05. *, P < 0.05; **, P < 0.01. FSW, female sex worker; Gzm, granzyme; MELC, multi-epitope ligand cartography; MFI, mean fluorescence intensity; UMAP, uniform manifold approximation and projection.

The epithelial sub-clustering of the CD8^+^ T cell cluster revealed five distinct sub-clusters numbered 4–8 (**Figure 6A**). The tissue-retention marker CD69 was elevated within sub-clusters 4–7, indicating high numbers of TRM CD8^+^ T cells, whereas sub-cluster 8 contained low levels of CD69 and was, thus, considered to comprise non-TRM CD8^+^ T cells. Sub-cluster 4 was further defined by elevated expression of the cytotoxic serine proteases granzyme (Gzm) A and GzmB, the transcription factor Eomesodermin (Eomes), and HLA-DR/DP/DQ, indicating the presence of activated cells. Additionally, sub-cluster 4 cells showed low expression of the epithelial markers E-cadherin and CD103, which are important for migration and retention within the epithelium and, therefore, were classified as submucosal activated cytotoxic CD8^+^ TRM cells. Sub-cluster 5 had a phenotype like that of sub-cluster 4 but exhibited elevated expression of CD103, suggesting the presence of CD8^+^CD103^+^ activated submucosal TRM cells. Sub-cluster 6 showed a marked elevation in CD161 levels, typically associated with mucosal-associated invariant T (MAIT) cells. However, as TCR Vα7.2 displayed scattered expression across the sub-clusters, it is likely that sub-cluster 6 is a mix of MAIT cells and less cytotoxic TRM CD8^+^ T cells. Additionally, sub-cluster 6 cells showed decreased expression of HLA-DR/DP/DQ and were thus considered to be inactive (**Figures 6B, C**). Finally, we detected decreased expression of the cytotoxic molecules GzmA and B in sub-cluster 7, along with high levels of both CD103 and E-cadherin; thus, these cells were identified as non-cytotoxic intraepithelial CD8^+^ TRM cells.

No differences were observed when comparing the frequencies of any of the CD8^+^ T cell sub-clusters between the two study groups. However, like the observed increase in leukocytes in HIV^+^FSWs, the total CD8^+^ T cell population in this group was increased 3.6-fold compared with the HIV^−^ group (**Figures 6D, E**). Quantification of individual CD8^+^ T cell sub-clusters revealed a significant HIV-associated increase in the distinct types of activated CD8^+^ TRM cells present in sub-clusters 4 and 5. Additionally, an HIV-associated increase in sub-cluster 8 was observed, which comprises non-TRM CD8^+^ cells (**Figure 6E**; **Supplementary Table S7**).

Collectively, our sub-clustering analysis revealed that the majority of CD8^+^ T cells present within the ectocervix are TRM cells. Furthermore, although we observed no HIV-associated difference in the frequency of any CD8^+^ T cell population between groups, we detected a significant increase in the number of both TRM and non-TRM CD8^+^ cells, indicating a local expansion of these cells and/or recruitment from the periphery.

### 3.9 HIV infection is not associated with alterations in CD4^+^ T cell populations

**Figure 7:**
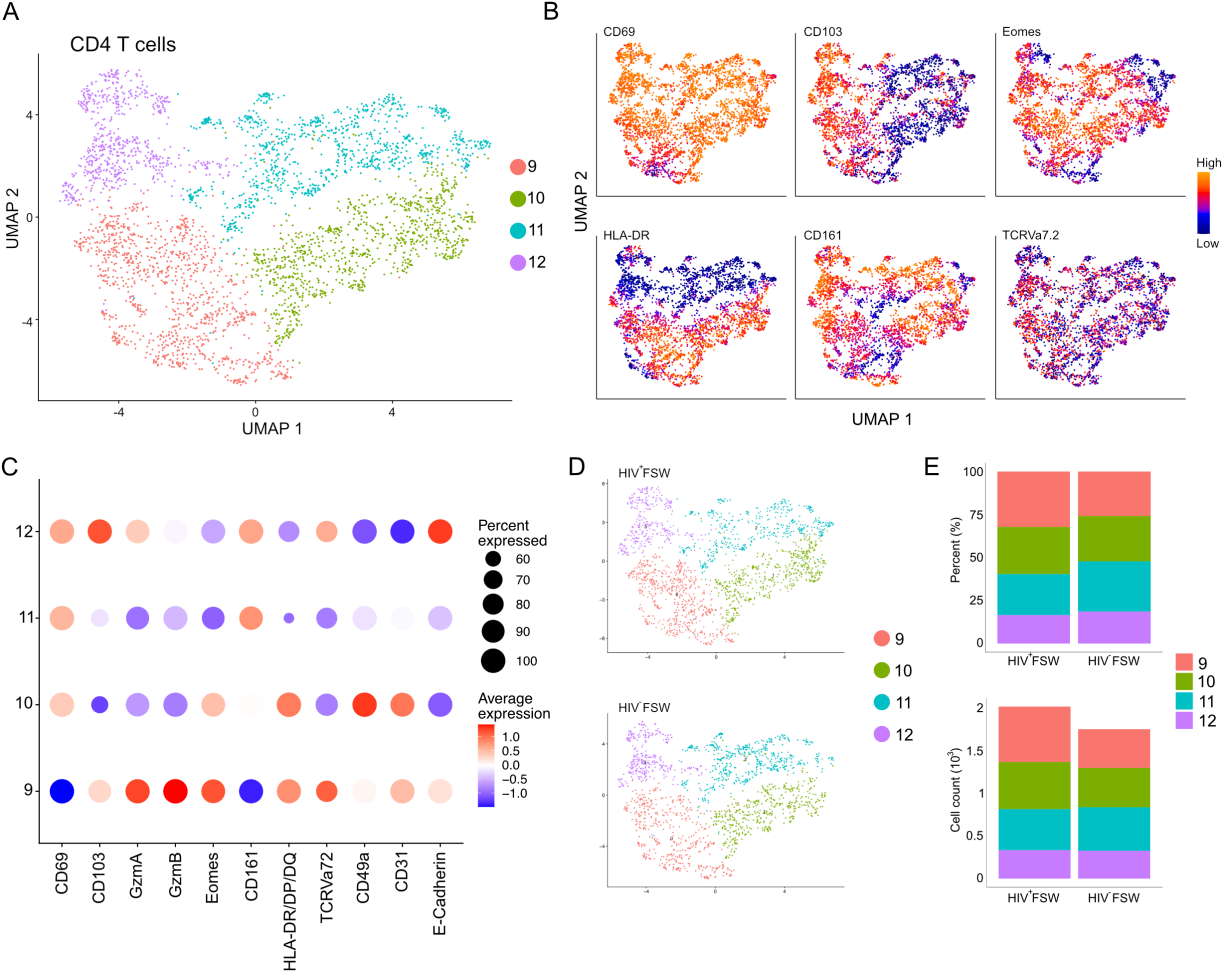
Cervical CD4^+^ T cell levels are unchanged in women with chronic HIV infection. **(A)** UMAP analysis followed by unsupervised clustering of ectocervical CD4^+^ T cells from MELC-stained tissue sections. **(B)** Feature plot visualizing cellular protein expression of CD69, CD103, Eomes, HLA-DR/DP/DQ, CD161, and TCR Vα7.2. **(C)** Dot plot visualizing MFI levels of individual markers within each CD4^+^ T cell sub-cluster. **(D)** UMAP plot showing the sub-clustering and CD4^+^ T cell prevalence within HIV^+^FSWs and HIV^−^FSWs. **(E)** Bar chart showing the frequencies (upper) and total cell counts (lower) of the CD4^+^ T cell clusters within the two study groups. Statistical significance was determined using the Mann–Whitney U test with significance set at P < 0.05. FSW, female sex worker; MELC, multi-epitope ligand cartography; MFI, mean fluorescence intensity; UMAP, uniform manifold approximation and projection.

Sub-clustering of the original CD4^+^ T cell cluster revealed four distinct sub-clusters, 9–12 (**Figure 7A**). Sub-cluster 9 was classified as CD4^+^ non-TRM cells (CD4^+^CD69^neg/low^) and showed high levels of HLA-DR, GzmA/B, Eomes, and CD103 expression, indicative of activated cells with a cytotoxic potential present in epithelium. Like CD8^+^ T cells, the remaining sub-clusters 10–12, representing the majority of CD4^+^ cells, expressed high levels of CD69 and were identified as CD4^+^ TRM cells (**Figures 7A–C**). All CD4^+^ TRM clusters expressed moderate levels of CD161, which is involved in secretion of interleukin (IL)-17, and, thus, they may represent T helper (Th)17 cells (44, 45). Sub-cluster 10 also exhibited elevated levels of CD49a, CD31, Eomes, and HLA-DR/DP/DQ and, therefore, likely contains activated submucosal CD4^+^ TRM cells. In contrast, given that cells in sub-cluster 11 expressed low levels of these markers, it likely contains inactive CD4^+^ TRM cells. Finally, sub-cluster 12 cells exhibited high levels of both CD103 and E-cadherin expression and were, therefore, classified as intraepithelial CD4^+^ TRM cells (**Figure 7C**). The relative proportions of each sub-cluster were comparable between the two study groups, and no differences in the number of cells within each cluster could be observed (**Figures 7D, E**; **Supplementary Table S7**). Thus, these results suggest that the CD4^+^ T cell populations remain relatively stable within the cervix of women chronically infected with HIV.

### 3.10 HIV infection is associated with an expansion of NK cells, macrophages, and dendritic cells in the ectocervix

**Figure 8:**
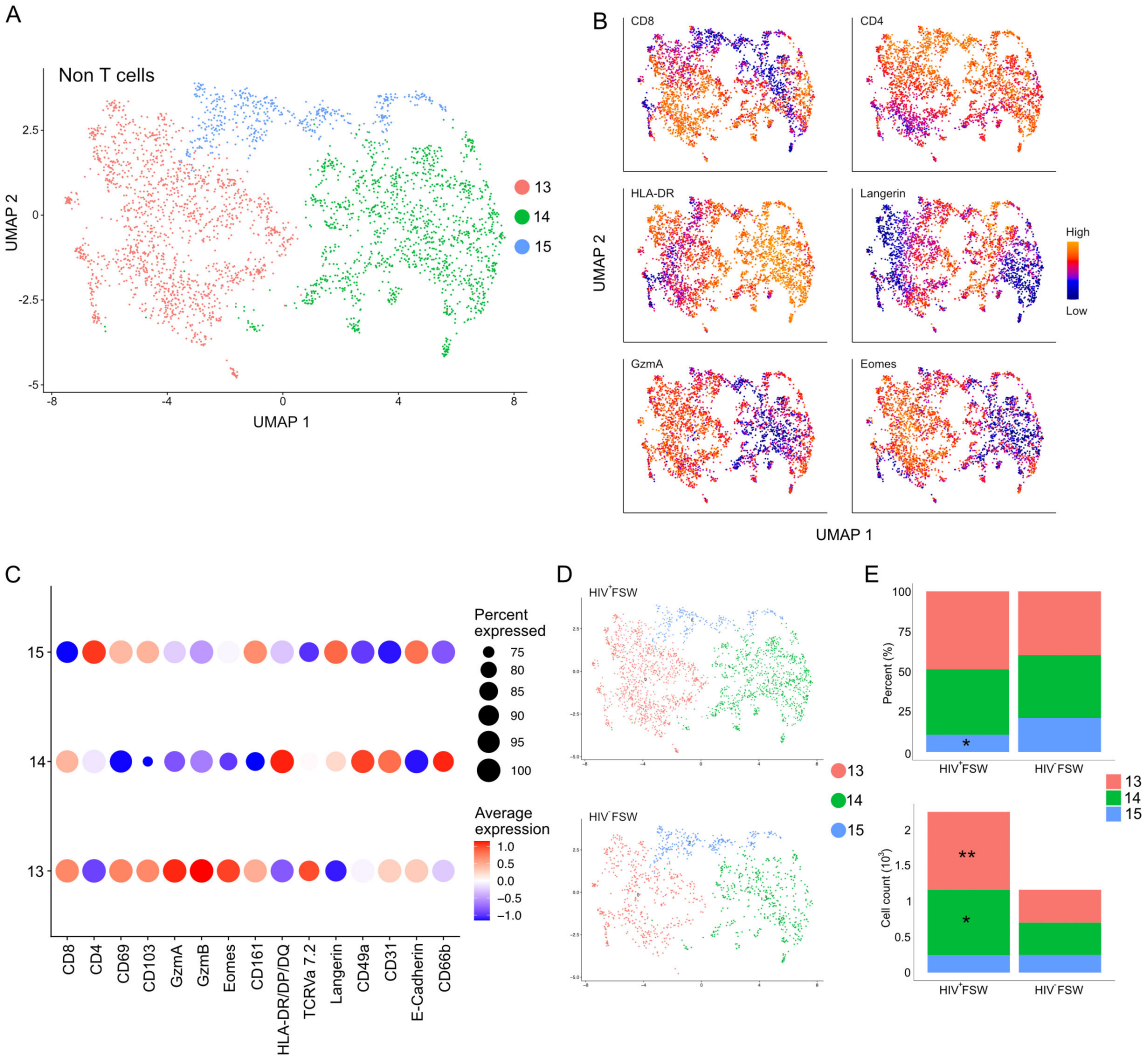
Increased cervical CD3^low/neg^ leukocyte levels are observed in women with chronic HIV infection. **(A)** UMAP analysis followed by unsupervised clustering of ectocervical CD3^low/neg^ leukocytes from MELC-stained tissue sections. **(B)** Feature plot visualizing cellular protein expression of CD8, CD4, HLA-DR/DP/DQ, Langerin, GzmA, and Eomes. **(C)** Dot plot visualizing MFI levels of individual markers within each CD3^low/neg^ leukocyte sub-cluster. **(D)** UMAP showing the sub-clustering and CD3^low/neg^ leukocyte prevalence within the HIV^+^FSWs and HIV^−^FSWs. **(E)** Bar chart showing the frequencies (upper) and total cell counts (lower) of the CD3^low/neg^ leukocyte clusters within the two study groups. Statistical significance was determined using the Mann–Whitney U test with significance set at P < 0.05. *, P < 0.05; **, P < 0.01. FSW, female sex worker; Gzm, granzyme; MELC, multi-epitope ligand cartography; MFI, mean fluorescence intensity; UMAP, uniform manifold approximation and projection.

Finally, we assessed the CD3^low^ and non-T cells identified by MELC. As cluster 3 was primarily characterized by co-expression of CD31 and CD49a, we focused our attention on cluster 2. Sub-clustering of this cluster revealed three distinct sub-clusters numbered 13–15 (**Figure 8A**). Sub-cluster 13 exhibited elevated levels of CD8 and likely contained both CD8^+^ T cells and NK cells. This population further showed increased expression of both granzymes, indicative of an activated cytotoxic phenotype, and displayed high levels of CD161, which is expressed on most NK cells, further suggesting their presence within this population. Sub-cluster 14 cells showed high-to-moderate expression of CD4 and high expression of HLA-DR/DP/DQ and were, thus, considered to be a mix of CD3^low^ CD4^+^ T cells and other CD4^+^ leukocytes, including macrophages and dendritic cells. Finally, sub-cluster 15 cells expressed high levels of both CD4, langerin, and E-cadherin and were classified as intraepithelial Langerhans cells (**Figures 8B, C**).

Similar to our findings with leukocytes and CD8^+^ T cells, we detected a 1.9-fold increase in CD3^low^ and non-T cells in tissue from HIV^+^FSWs. Comparing the sub-cluster frequencies between study groups revealed an HIV-associated decrease in the frequency of sub-cluster 15 comprised primarily of Langerhans cells, with no differences in the frequencies of the remaining clusters. In contrast, quantification of the number of cells within each sub-cluster revealed a significant HIV-associated increase in cells comprising both sub-cluster 13 and 14, which contain CD4^+^ and CD8 T cells, respectively, as well as NK cells, macrophages, and dendritic cells (**Figures 8D, E**; **Supplementary Table S7**). Thus, in addition to an expansion of CD8^+^ T cells, our results indicate an HIV-associated increase in non-T cells within the ectocervix, including NK cells, macrophages, and dendritic cells, providing further evidence for broad immune dysregulation in chronically infected women.

## 4 Discussion

In the present study, we performed an in-depth exploration of the transcriptional and cellular dynamics in ectocervical tissue samples of women living with HIV infection. To our knowledge, this is the first time the cutting-edge spatial transcriptomics methodology has been employed to map the ectocervical transcriptome of HIV-infected women, providing unprecedented insight into the spatial distribution of gene expression in these individuals. We then expanded on these analyses by using MELC, a massive multiplex fluorescent staining technology, to explore the intricate immunological milieu of the ectocervix at a cellular level, providing a comprehensive assessment of its immune landscape. Our gene expression data reveal a pronounced HIV-associated induction of immunoregulatory genes, along with activation of cellular immune responses and IFN signaling. Interestingly, a tissue-wide B cell response was also evident from the induction of several genes encoding for different antibody isoforms. We also observed structural remodeling of both the epithelium and submucosa within the HIV-positive samples. Similarly, MELC analysis revealed immune activation associated with HIV infection, as exemplified by an increased density of leukocytes and driven primarily by CD8^+^ T cells.

Research directed at assessing tissue-specific immune responses using samples from the FRT is associated with considerable logistical and ethical challenges and thus investigations have primarily been made using other mucosal sample sources (19–22). Results on the prevalence of cell populations can however vary when comparing data from microscopy and e.g. mucosal single-cell suspensions (46). Crucially, the use of tissue samples preserves the spatial information and phenotypic expression of the immune cells, providing contextual insights. A pivotal finding of our study was the presence of a tissue-wide upregulation of B cell–related genes in HIV^+^ samples. This aligns well with increased protein levels of both IgG and IgA in cervicovaginal secretions from HIV-1-infected versus uninfected controls in a cohort of asymptomatic African women (47). Within the epithelium and the more immunologically active and epithelial-adjacent submucosal clusters, we detected a convincing upregulation of several antibody-related genes including *IGHG1–4*. In addition, several light chain genes were upregulated lending physiological relevance to the upregulation of *IGHG* related genes. We further observed the upregulation of both *IGHA1* and *IGHA2* within clusters more distally located from the epithelium. Early in the HIV epidemic, systemic hypogammaglobulinemia and polyclonal B cell activation were reported in infected individuals, with histological studies demonstrating a loss of germinal centers in secondary lymphoid organs (48–50). Moreover, systemic plasmablasts, a relatively rare cell population during homeostatic conditions, are increased during HIV infection, and their predominant production of IgA is decreased in favor of IgG, likely due to polyclonal activation leading to production of non-HIV-specific antibodies (51). Similarly, mucosal studies of the GI tract have also reported a loss of germinal centers in response to HIV infection, which is thought to result from the dysregulation and expansion of Th follicular cells and a bias toward IgG-producing cells (52).

B cells within the FRT are rare, representing less than 5% of all immune cells, with plasma cells primarily located within the cervix (53, 54). Although few studies have investigated the effects of HIV infection on the B cell population within this tissue, one report noted an increased prevalence of B cells in cervicovaginal lavage samples from HIV-infected women coupled with an increased predisposition toward IgG secretion (55). Our spatial transcriptomics data reveal a strong upregulation of IgG production, thus mirroring what has been previously shown both systemically and within the GI tract. Although we also observed an upregulation of *IGHA* associated with chronic HIV infection, this effect was less pronounced and more sporadically expressed than that detected for *IGHG*. Thus, we infer that the observed IgG induction in our study is part of a dysregulated cervical B cell response, potentially mediated by strong plasmablast induction. However, as has been demonstrated within other compartments, these cells are likely polyclonal within the cervix and, thus, fail to control HIV replication within this tissue (51). Alternatively, increased antibody production may result from an HIV-associated activation of innate marginal zone B cells, given that we also observed the upregulation of genes relating to this population, including *MZB1* (56). Marginal zone B cells possess innate-like properties, allowing them to rapidly mount a response to pathogens. Interestingly, this population is increased within HIV^+^FSW compared to their HIV^−^ counterparts, highlighting a potential role in B cell dysregulation (57). Finally, it is also possible that the observed B cell dysregulation within the FRT is not a direct result of HIV infection but rather occurs secondarily due to sustained inflammation, as a dysregulated IgG-secreting plasma cell response has been demonstrated during several other chronic inflammatory conditions (58).

Our spatial transcriptomic data further show an induction of multiple T cell–related genes across several clusters present within both the epithelial and submucosal compartments. These findings were further verified by immunofluorescence staining. Moreover, although a general increase in leukocytes in response to chronic HIV infection was observed within the FRT, our data indicate that T cells are the predominant population driving this observed increase. Comparing the frequencies of the two major T cell populations (i.e., CD4^+^ and CD8^+^ cells) further revealed that the increase is mediated by CD8^+^ T cell induction, which is indicative of the dysregulated immune response typically observed in HIV infection. Interestingly, when sub-clustering the CD8^+^ T cells, we detected an increase in both TRM and non-TRM populations, suggesting an expansion of local TRM cells and/or the potential recruitment of circulatory CD8^+^ T cells into the mucosa. Although studies of TRM T cells in humans are becoming more frequent, such investigations are still limited within the FRT and even rarer in the context of HIV infection. Thus, the precise function of TRM T cell immunity in HIV has not yet been fully elucidated. Moreover, the available studies suggest that their role differs depending on the tissue (59–61).

In mice, intraepithelial CD8^+^ TRM T cells display a cytotoxic phenotype, whereas their counterparts in humans have been reported to show less cytotoxic activity (62, 63). As such, TRM T cells in humans are proposed to operate more as innate-like sensors that recruit other immune cells to the site of infection (64). Here, however, we observed an increased prevalence of CD8^+^ TRM T cells expressing granzymes GzmA and GzmB in ectocervical samples from the HIV-infected group. Of note, and consistent with this finding, Reuter et al. have reported an upregulation of GzmB in secondary lymphoid organs of HIV-infected individuals (65). Unfortunately, because we lack perforin staining, we cannot specifically define these cells as cytotoxic. However, the data collectively suggest that sustained immune activation or repeated antigen exposure could trigger a cytotoxic profile within CD8^+^ TRM T cells.

CD4^+^ T cell depletion is a hallmark of HIV infection and has been observed within the blood, lymphoid organs, and GI tract of infected individuals. However, the extent of this depletion within the FRT remains disputed, with studies on cervicovaginal lavage fluid showing a decreased prevalence of Th17 cells while microscopy studies assessing cervical tissue indicating a stable CD4^+^ cell population (66, 67). In the present study, we observed no differences in CD4^+^ T cell prevalence between the two study groups, indicating that, unlike the GI tract, the T cell population within the FRT remains stable during chronic HIV infection. Furthermore, although an HIV-associated decreased frequency of CD4^+^ T cells was noted, this reduction is likely due to the expansion of CD8^+^ T cells and other leukocytes rather than a depletion of CD4^+^ T cells, as evident from the stable numbers of CD4^+^ T cells detected. Similar to CD8^+^ T cells, the CD4^+^ T cells were predominantly TRM cells, as defined by high expression of CD69. This population was recently found to be a critical viral reservoir within the FRT and was also shown to be preferentially infected in *ex vivo* studies (68). Consistent with prior studies, we also found that CD4^+^ T cells were the predominant CD4^+^ cell population in the ectocervix (69). Given the longevity and proportion of CD4^+^ TRM T cells, their contribution to viral persistence might be of substantial importance. Unfortunately, markers to clearly distinguish the Th17 population were not included, and thus, we cannot exclude the possibility of a preferential depletion of this population within the ectocervix of HIV-infected women. We further note that, like the T cell population, no differences in the amount of Langerhans cells were detected, indicating that this population also remains stable during chronic HIV infection. We did, however, observe an HIV-associated increase in CD3^low/neg^ CD4^+^ cells, which is indicative of an expansion of macrophages and/or dendritic cells.

Our gene expression data further reveals the HIV-associated induction of several IFN-related genes, which are essential for viral clearance. However, a dysregulated IFN response has been proposed as a central mechanism for the systemic immune activation present in HIV-infected individuals. Thus, aberrant IFN signaling is a likely contributor to the cervical immune activation observed in the present study (70, 71). Additionally, given the key role of IFN signaling in T cell regulation, a seemingly dysregulated IFN response is expected to play a role in the T cell induction and immune dysregulation observed in ectocervical tissue from participants with HIV. Of note, IFN signaling has also been implicated in B cell activation and plasma cell differentiation (72). Thus, dysregulated IFN responses may further contribute to the B cell activation and *IGHG* production observed in those with chronic HIV.

Crucially, the use of spatial transcriptomics allowed us to assess spatially restricted gene expression patterns and assign discernable functions associated with each identified mucosal layer. The superficial epithelial layer, located toward the cervicovaginal lumen, showed an HIV-associated upregulation of genes involved in mucosal healing and viral-induced inflammation, whereas a strong induction of IFN-related genes was detected in the upper intermediate layer, possibly due to viral replication. The lower intermediate and basal layers similarly displayed an HIV-associated upregulation of several inflammatory genes, specifically those related to cellular immunity, in accordance with the spatial localization and HIV-associated induction of T cells. Downregulated genes included those involved in terminal keratinocyte differentiation within the upper intermediate and superficial layers and epithelial stability and function within the lower intermediate and basal layers. Similarly, prior studies have reported an HIV-associated epithelial disruption *ex vivo* following acute viral exposure (16, 17). Moreover, the most prominent differentially altered pathways associated with HIV were developmental processes for both the epithelium and submucosa. Collectively, these findings indicate that HIV infection and subsequent chronic inflammation may promote structural remodeling of the submucosa, potentially impacting disease pathogenesis and immune dysfunction and warranting further investigation.

In addition to the use of unique tissue sample material and cutting-edge methodologies, a key strength of our study is the inclusion of sex workers as participants, given that these individuals are part of a vulnerable group with an increased prevalence rate of HIV infection. Critically, an improved understanding of HIV pathogenesis in this unique cohort may offer valuable insights into HIV transmission and the potential indirect effects of an HIV-mediated dysregulated immune response on other prevalent genital infections, such as human papillomavirus (HPV) and herpes simplex virus (HSV)-2. However, we note that this study has several limitations. First, although our sample size is similar to that used in previous studies utilizing similar methods, the small sample size may not fully reflect the larger study population (73). Moreover, even though none of the participants had visible cervical dysplasia, neither HPV DNA nor pap-smear diagnostics was performed. It would also have been beneficial to perform scRNA-seq on biopsies collected in the same cohort to attain a reference dataset that better reflects the generated spatial transcriptomics data. Additionally, the MELC panel was predefined to include mainly T cell-related genes. As a significant upregulation of humoral genes was observed in the current study, it would have been beneficial to include B cell markers in the panel. Markers to distinguish macrophages and dendritic cells could also have been used to further differentiate the CD4^+^ cell populations. Since CD4^+^ T cells can downregulate the CD4 receptor upon HIV infection (74–76), staining for viral particles would have avoided possible miss-classification of HIV infected CD4^+^ T cells as CD8^+^ T cells. We employed a data-driven approach by performing unsupervised hierarchical clustering based on protein expression levels within the ectocervical leukocytes from both study groups. However, we are aware that due to the need for cell segmentation, there are cross-contamination issues for adjacent or tiled cells. Finally, given that only 17 markers were used, we can only provide a reasonable approximation for the clustering of specific leukocyte populations. Similarly, although CD69 is used as a canonical TRM cell marker, an inclusion of additional TRM markers would have been beneficial to further elucidate this population. In future studies it would be highly informative to perform an integrated analysis of spatial transcriptomics and MELC data to deepen the insights into spatial protein expression patterns. Other important aspects to explore include the temporal variation in gene expression relative to the time since HIV infection and the interplay between these immune changes and the microbiome. Identifying specific cells that harbor HIV could also provide a more comprehensive understanding distinguishing tissue-specific changes from systemic immune activation.

In total, the results of the present study advance our understanding of the local genital immune response in women living with HIV infection. Our findings may also provide insights into HIV transmission and the increased prevalence of other sexually transmitted infections in similar populations due to the dysregulated epithelium and immune response. Furthermore, given the critical importance of a functional mucosal immune response for defense against viral pathogens, the results of this study could have implications for future strategies directed at preventing HIV infection.

## Data Availability

The clinical characteristics of the study participants, and the raw counts of the spatial RNA sequencing, are available at the Gene Expression Omnibus (GEO) public repository (accession ID: GSE217237). The raw RNA sequencing data, as well as some additional sociodemographic and clinical characteristics of the study participants, cannot be held in a public repository because of the sensitive nature of such personal data. Requests for data access can be made to the Karolinska Institutet Research Data Office (contact via rdo@ki.se). Access will be granted if requests meet the requirements of the data policy. All scripts used for the spatial RNA sequencing can be found on GitHub (https://github.com/vildeka/Spatial_DMPA). The bioimage analysis workflow is also available at GitHub (https://github.com/MathiasFranzenBoger/MELC-leukocyte-analysis).
