# Supplementary material for: Spatial transcriptomics and in situ immune cell profiling of the host ectocervical landscape of HIV infected Kenyan sex working women
Source: Front Immunol. 2024 Dec 2;15:1483346. doi: 10.3389/fimmu.2024.1483346 (PMC11646855; doi:10.3389/fimmu.2024.1483346)
Supplement: Supplementary file 8 [file DataSheet1.pdf]

Supplementary Figures

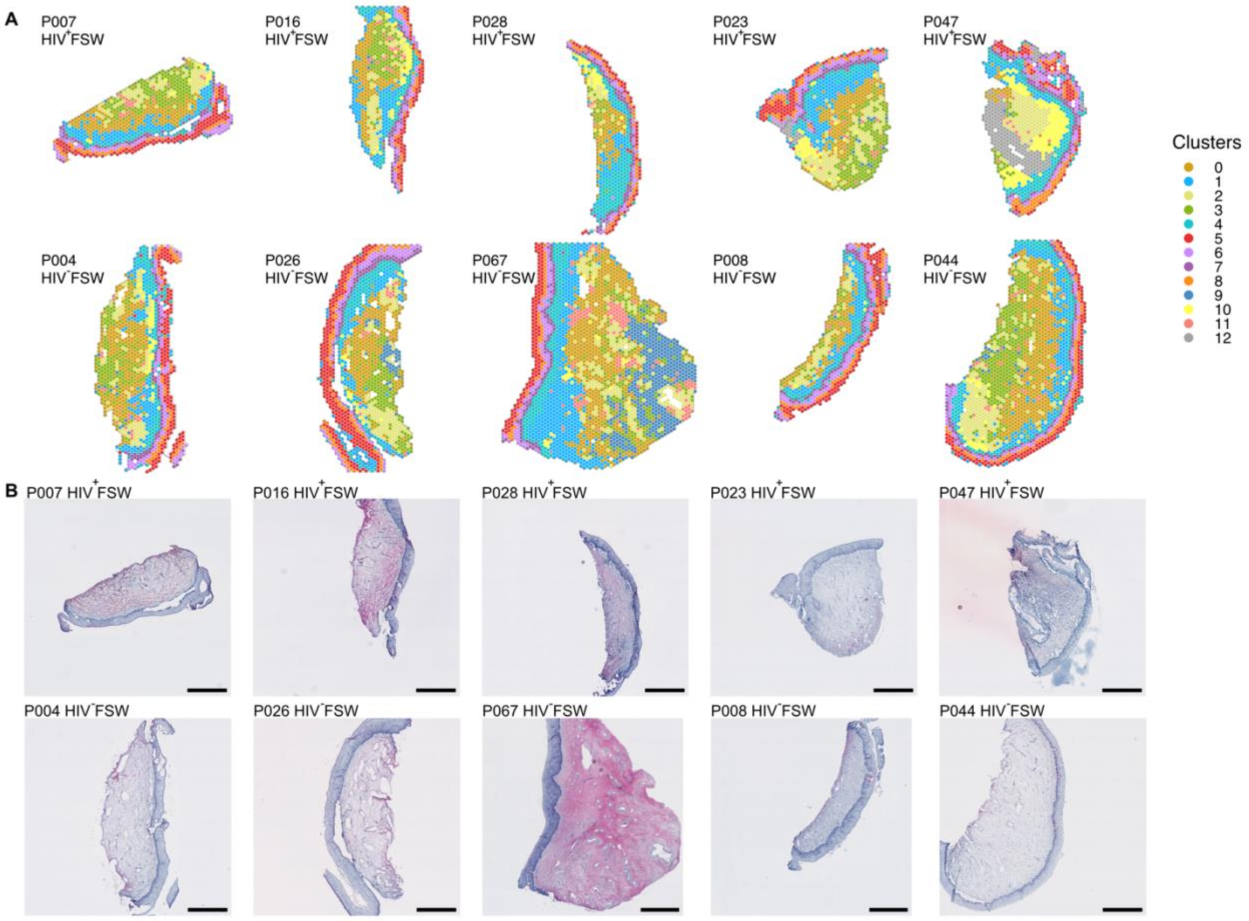

Supplementary Figure S1. Unsupervised clustering and H&E staining of full tissue sections from all study participants. A) Unsupervised clustering using UMAP plotted on tissue. Classification by unsupervised Louvain clustering determined from UMAP analysis of gene expression profiles plotted on tissue from HIV^{+}FSW and HIV^{-}FSWs, revealing a total of 13 ectocervical clusters. B) H&E staining of all study participants. Scale bars represent 500 μm. H&E; Hematoxylin and eosin. FSW; Female sex worker; UMAP, uniform manifold approximation and projection.

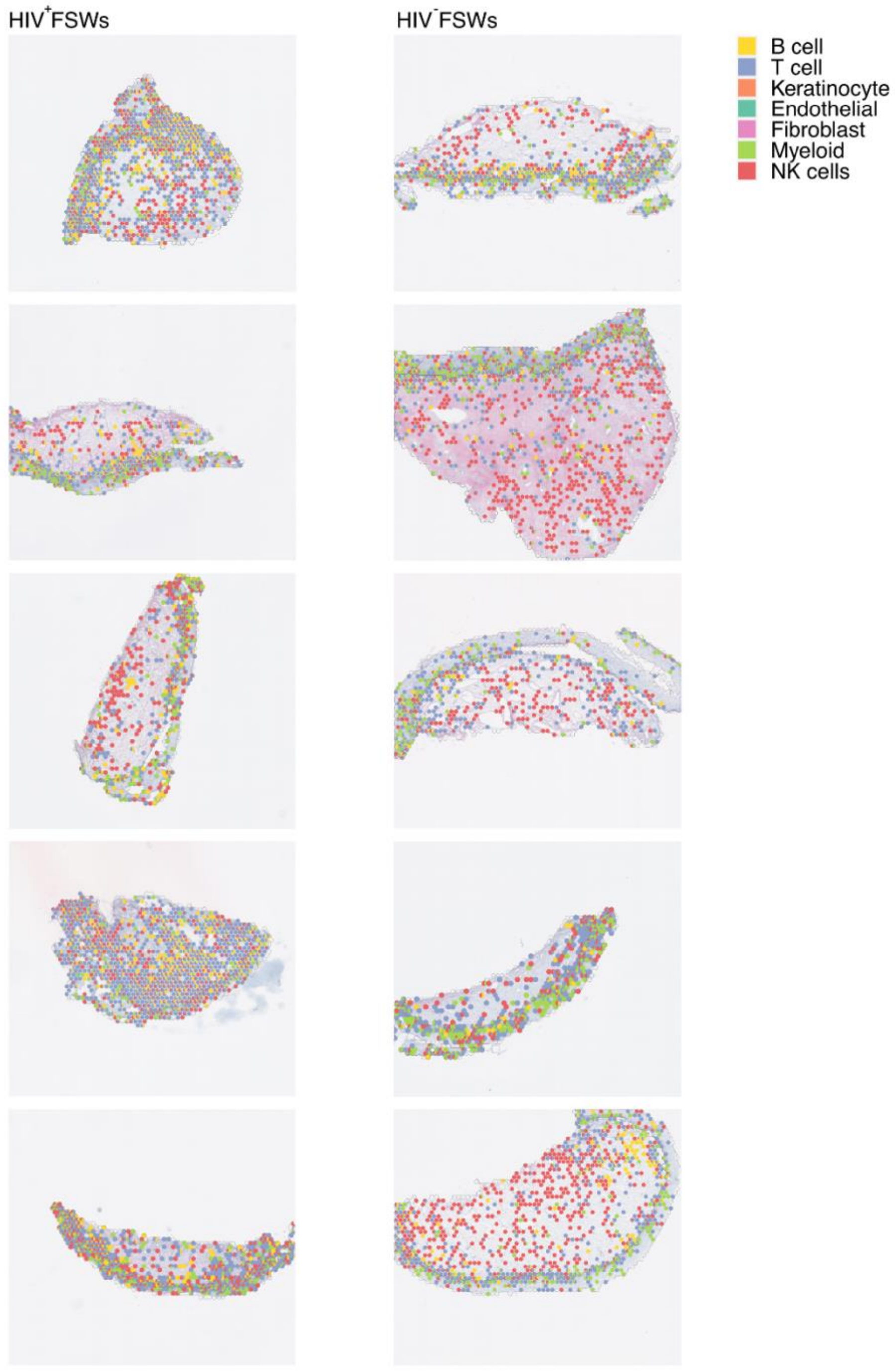

Supplementary Figure S2. Visualization of the immune cell populations present within the ectocervix of all study participants. Relative gene expression levels of the select cell markers in every spot plotted on tissue with keratinocyte- and fibroblast-related genes excluded to better visualize immune cell distribution in tissue of HIV⁺FSWs and HIV⁻FSWs. FSW, female sex worker.

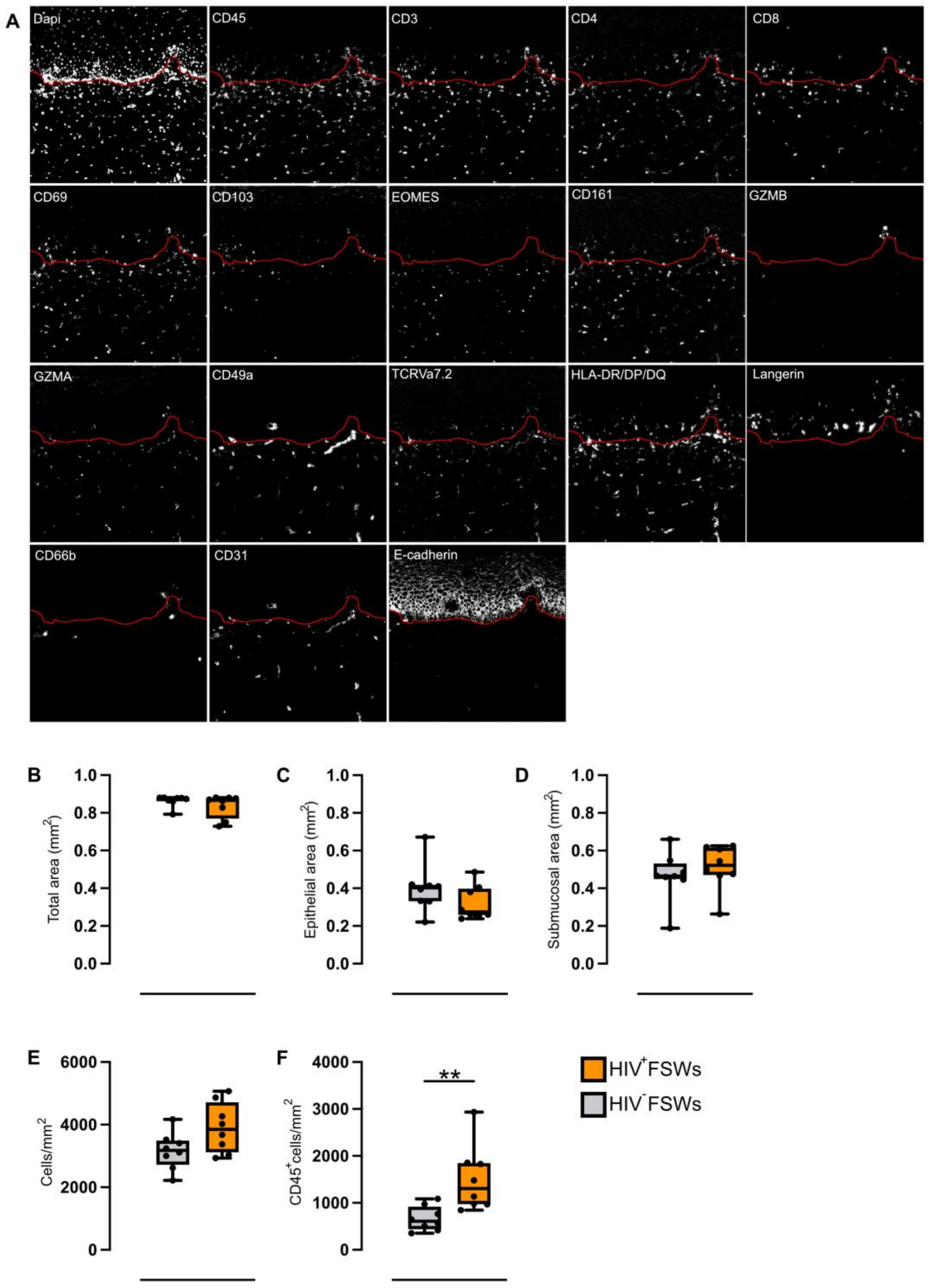

Supplementary Figure S3. MELC panel and leukocyte density: A) Overview of the panel used for MELC run within the ectocervix of HIV^{+}FSWs and HIV^{-}FSWs. Each image depicts the same FOV which was sequentially stained with the depicted antibodies. The epithelium (upper) and submucosa

(lower) are separated by the red line. The (B) total, (C) epithelial and (D) submucosal area was calculated and compared between study groups. The (E) cell and (F) leukocyte density (cells/mm^2) were calculated. Boxplots indicate the median and IQR, whereas whiskers show the full range of individual values plotted. Statistical significance was determined using the Mann–Whitney U test with significance set at $P < 0.05$. **, $P < 0.01$. Brightness and contrast were adjusted for visualization purposes. FOV, field of view; FSW, female sex worker; IQR, interquartile range; MELC, multi-epitope ligand cartography.
